# Production of Red Beetroot (*Beta vulgaris* L.) Pestil as an Alternative Healthy Snack: Effects of Traditional, Thermosonication, and Microwave Pretreatments on Physicochemical, Sensorial, Nutritional, and Chemometric Profiles

**DOI:** 10.3390/foods14101784

**Published:** 2025-05-17

**Authors:** Neslihan Ersoyak, Busra Acoglu Celik, Elif Koc Alibasoglu, Erturk Bekar, Taha Turgut Unal, Ersin Yetan, Senem Kamiloglu, Arzu Akpinar Bayizit, Omer Utku Copur, Irmak Aral Baskaya, Perihan Yolci Omeroglu

**Affiliations:** 1 Food Engineering Department, Faculty of Agriculture, Bursa Uludag University, Gorukle Campus, 16059 Bursa, Türkiye; nersoyak@gmail.com (N.E.); busraacoglu@gmail.com (B.A.C.); elifkoc0894@gmail.com (E.K.A.); erturk@uludag.edu.tr (E.B.); tahaunal@uludag.edu.tr (T.T.U.); ersinyetan@gmail.com (E.Y.); skamiloglu@uludag.edu.tr (S.K.); abayizit@uludag.edu.tr (A.A.B.); ucopur@uludag.edu.tr (O.U.C.); aralllirmak@gmail.com (I.A.B.); 2 Science and Technology Application and Research Center (BITUAM), Bursa Uludag University, Gorukle Campus, 16059 Bursa, Türkiye

**Keywords:** red beetroot, pestil, healthy snack, thermosonication, microwave, phenolic, betalain, mineral, bioactive compounds, hydroxymethylfurfural

## Abstract

Red beetroot (*Beta vulgaris* L.) is a nutritionally rich root vegetable. It is a potential alternative raw material for pestil, a traditional fruit-based snack. This study aimed to develop a healthy beetroot-based pestil using traditional boiling (95 °C) and novel pretreatment methods, including thermosonication and microwave processing, with and without additional concentration steps. The effects of these methods on heat treatment period, hydroxymethylfurfural (HMF) formation, and the physicochemical, sensorial, nutritional, and chemometric profiles of pestils were evaluated. The beetroot-based snack formulated in this study was hedonically acceptable (≥5/9) and rich in essential minerals (Ca, K, Na, P, Mg) and trace elements (Zn, Fe, Mn), as determined by inductively coupled plasma–mass spectrometry. Total antioxidant capacity (CUPRAC) ranged from 113.11 to 870.78 mg Trolox^®^ equivalent/100 g dry matter (DM). Total phenolic, flavonoid, and betalain contents varied between 220.6–313.8 mg gallic acid equivalent/100 g DM, 365.08–517.46 mg rutin equivalent/100 g DM, and 314.40–488.66 mg/kg, respectively. Major flavonoids identified and quantified included epicatechin, rutin, isoquercitrin, taxifolin, and quercetin, while major phenolic acids identified were chlorogenic acid, ferulic acid, caffeic acid, o-salicylic acid, p-coumaric acid, and vanillin, using liquid chromatography–electrospray tandem mass spectrometry. Reducing the soluble solids content of the pestil pulp from 40 to 20 Brix degrees, in combination with thermosonication and microwave treatments, significantly shortened the processing time by 10–67%. This approach also reduced the HMF content to the limit of quantification (LOQ). Pretreatment methods significantly (*p* < 0.05) affected the levels of minerals and bioactive compounds in the pestils. These findings highlight the importance of process optimization to improve overall safety and the nutritional quality of the pestil.

## 1. Introduction

Today, consumers are increasingly interested in functional foods to improve their dietary habits and prevent chronic diseases. In this context, fruits and vegetables are regarded as functional foods due to their bioactive components. In recent years, red beetroot (*Beta vulgaris* L.) has been recognized as a valuable alternative raw material for functional food formulations due to its rich nutritional compositions and health benefits [1,2]. Red beetroot, belonging to the Amaranthaceae family, contains valuable compounds, such as carotenoids, glycine betaine, betacyanins, folates, betanin, and polyphenols, including flavonoids and phenolic acids. It is also rich in dietary fiber, vitamins A, C, and B-group vitamins, in addition to essential minerals, such as sodium, calcium, iron, phosphorus, potassium, magnesium, and zinc. The characteristic red color of beetroot is primarily attributed to betalain pigments, such as betanin and vulgaxanthin I [3], with their concentrations varying among different beetroot varieties. Red beetroot is generally consumed as fresh, while it is also commonly processed into juice, pickles, powder, food supplements, and natural food colorings [2,4,5,6,7]. However, there are currently no commercially available red beetroot-based pestil products, nor any scientific studies investigating its physicochemical, sensory, and nutritional quality as a functional snack.

Traditional pestil (fruit leather) production varies regionally; however, the general process involves pureeing cleaned and sorted fruits or vegetables with a homogenizer. To facilitate texture softening, a short boiling step is generally applied at 95 °C before pureeing. Depending on the product, seeds and peels are removed through straining. This is followed by a thickening (concentration) process carried out at 100–110 °C until the mixture reaches a target soluble solids content (measured by the degree Brix, °Bx) [8,9,10,11,12]. Although current pestil standards (28–32) do not specify a target °Bx value for the concentration step, traditional practices generally aim for around 40 °Bx [8,9,13,14,15,16]. However, several studies have reported that the heat treatment process may be terminated after a shorter period with lower concentrations, typically at 20 °Bx [10,11]. During the concentration process, textural modifiers or flour (approximately 5–15 g per 100 g of dry matter) are incorporated. Additionally, optional sugar derivatives may be added to promote starch gelatinization. The resulting viscous, gelatinized mixture, known as *herle*, is then uniformly spread into thin layers and subjected to controlled drying to form pestil [8,9].

To maintain structural integrity in pestil production, various thickeners—including wheat starch, corn starch, carob gum, Arabic gum, carboxymethyl cellulose, and sodium alginate—are incorporated at different concentrations [8,12,17,18]. Tapioca starch is another potential thickener, widely utilized in gluten-free products, such as cakes and noodles, due to its rapid swelling properties and high water retention capacity [12,19,20,21]. However, no studies in the existing literature have reported the use of tapioca starch in pestil production. The use of alternative sweeteners is also essential in the development of healthier snack products. In this context, apple serves as a natural sweetener alternative suitable for fruit pulp formulations.

Pretreatment methods in fruit and vegetable processing improve quality, shelf-life, and efficiency. Conventional methods, like blanching (or boiling) and alkali immersion, have drawbacks, such as nutrient loss and high energy use. Emerging technologies, including cold plasma, pulsed electric fields, ultrasonication, thermosonication, and microwaves, offer better preservation of nutrients and improved processing efficiency [22]. Ultrasonic (US) technology is a promising non-thermal pretreatment method in food processing, such as drying and extraction [23,24]. Ultrasound operates at frequencies of 20 kHz or higher, generating cavitation and fluid movement, which forms microchannels within plant tissues. As a pretreatment before drying, ultrasound enhances drying efficiency, promotes moisture movement, increases mass transfer, reduces drying time and energy consumption, and preserves heat-sensitive bioactive compounds, while retaining the nutritional, textural, and sensory properties of the food [25,26,27,28,29]. Studies in the literature have shown that combining ultrasonic treatment with heat and/or pressure enhances the inactivation of enzymes (such as polyphenol oxidase and peroxidase) and microorganisms, while also improving the stability of bioactive compounds. This method, known as thermosonication, achieves higher inactivation rates and can reduce processing time compared to ultrasound and heat treatment alone. During thermosonication, cavitation induced by ultrasound disrupts the structure of proteins and enzymes by breaking hydrogen bonds and modifying Van der Waals interactions, ultimately leading to the loss of enzymatic activity [25,30]. Microwave (MW) processing operating at frequencies between 300 MHz and 300 GHz is widely used in the food industry for its rapid and efficient heating, safety, and nutrient preservation [22]. MW heating is based on dielectric heating, where polar molecules in the food absorb electromagnetic energy and convert it into heat. This results in uniform heating throughout the product and creates an internal pressure gradient that moves moisture toward the surface. As a pretreatment before drying, it improves the drying efficiency, product quality, and retention of antioxidants and phenolics [31,32]. US and MW are more cost-effective and easier to implement in the food industry compared to pulsed electric field (PEF) and cold plasma methods. They offer benefits, such as lower equipment costs, energy efficiency, and easier integration into existing systems. In contrast, PEF and cold plasma require higher capital investment and technical expertise, which can limit their scalability [33,34]. In pestil production, an initial preheating step is typically carried out by boiling in an open vessel at approximately 95 °C, followed by a concentration step. However, no studies have yet reported the application of novel pretreatment methods, such as thermosonication and microwave treatments, in pestil production.

Hydroxymethylfurfural (HMF), found in various food products including pestils, exhibits both beneficial (antioxidant, anticarcinogenic, antiallergenic, anti-inflammatory, and antimicrobial) and detrimental (carcinogenic, genotoxic, mutagenic, and organotoxic) health effects. HMF is formed as a result of caramelization and Maillard reactions that occur at elevated temperatures, serving as an indicator of thermal processing in food products [8,9,10,12]. According to pestil standards released by the Turkish Standards Institute, the HMF concentration should not exceed 50 mg/kg [35,36,37,38]. While heat treatment improves texture and preservation, it can also result in HMF formation, which may negatively impact the nutritional and sensory qualities of the product. Thus, it is crucial to control heat treatment conditions and duration to reduce HMF formation and maintain the desired characteristics of the pestil [8,9,10,12]. Consequently, it is essential to investigate methods to shorten the duration of heat treatment processes in pestil production.

In light of the current knowledge and literature gaps, this study aimed to develop a healthy beetroot-based pestil using traditional boiling (95 °C) and novel pretreatment methods, including thermosonication and microwave, with and without additional concentration steps. The effects of these methods on heat treatment period, HMF formation, and the physicochemical, sensorial, nutritional, and chemometric profiles of pestils were evaluated.

## 2. Materials and Methods

### 2.1. Materials

The red beetroots (*Beta vulgaris* var. *conditiva*) and apples (*Malus domestica* cv. Starkrimson Delicious*) used in this study were obtained from a local market in Bursa Province, Türkiye. Prior to processing, the fruits and vegetables were stored under refrigerated conditions at 4 ± 0.5 °C for one month. Tapioca starch was purchased from TMS Organik Gıda San. Tic. Ltd. Şti (Antalya, Türkiye) via Talya Foods through an online marketplace.

### 2.2. Pestil Production

Preliminary trials were conducted to optimize the pestil formulation by testing different ratios of apple and red beetroot, as well as selecting an appropriate thickening agent, including tapioca starch. Additionally, key production parameters, such as the boiling method (open vessel, thermosonication, and microwave), concentration duration and target Brix levels (40, 20, and 15 °Bx), and drying temperature were systematically evaluated. To determine the final formulation and critical production parameters, the overall acceptability of pestils produced under different conditions was assessed by 10 trained panelists using a 9-point hedonic scale (1: dislike extremely, 9: like extremely). The final formulation and experimental design options that achieved an average score above 7 were selected for further analysis in the scope of this study. Based on those trials, the optimized pestil formulation contained 47% red beetroot, 47% red apple, 5.8% tapioca starch, and 0.2% citric acid by weight.

**Figure 1:**
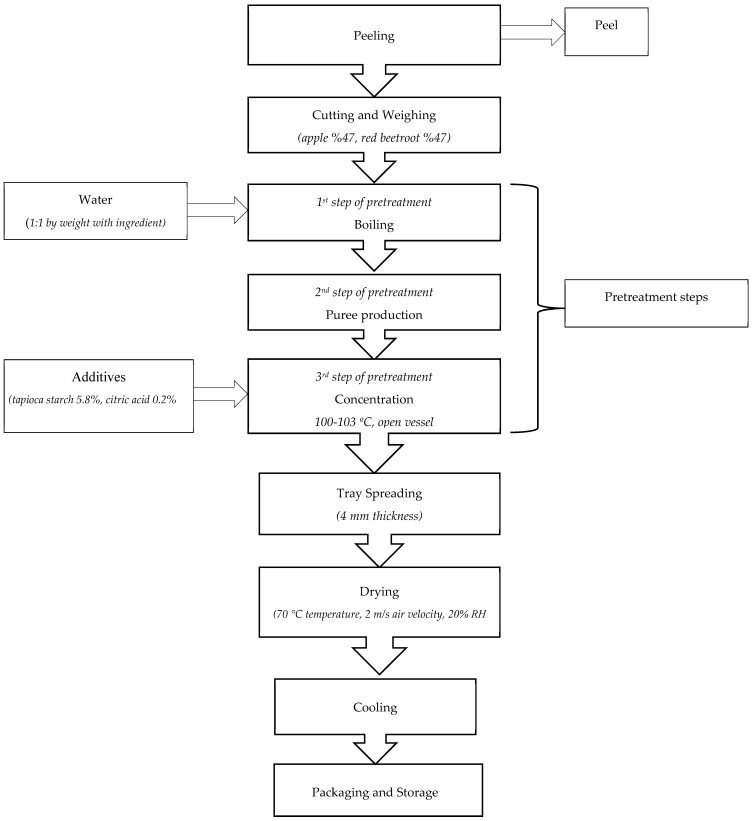
Flowchart for red beetroot pestil production.

**Figure 2:**
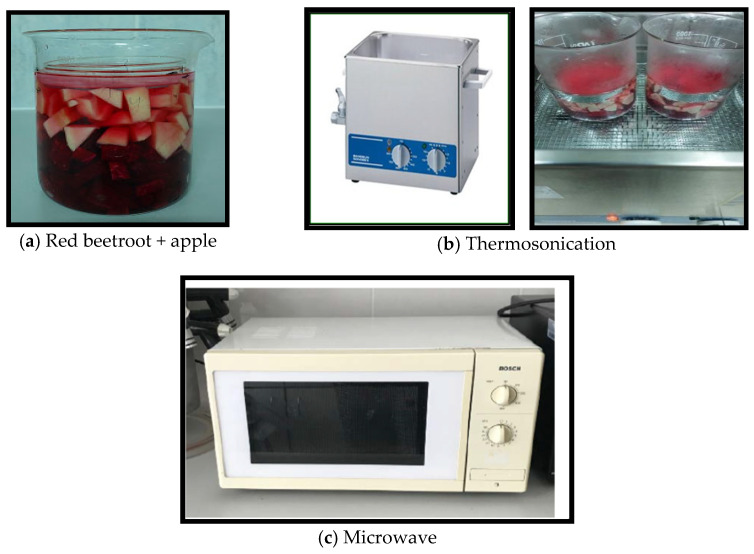
Thermosonication and microwave equipment.

Details of the pretreatment conditions and the production flowchart are provided in Table 1 and Figure 1, respectively. Thermosonication was performed using an ultrasonic bath (Bandelin Sonorex Super RK 510 H, Bandelin Electronic GmbH & Co., KG, Berlin, Germany) operating at a fixed frequency of 35 kHz and an effective ultrasonic power of approximately 640 W. The system generates continuous ultrasound waves (100% duty cycle) and is equipped with an integrated heating element capable of maintaining temperatures at 80 °C. The internal tank dimensions are 300 mm (length) × 240 mm (width) × 150 mm (depth), allowing for uniform sound wave propagation. Samples were placed in a sealed glass beaker (1000 mL) and positioned in the center of the bath (Figure 2), which was filled with distilled water as the coupling medium. MW pretreatment was conducted using a domestic microwave oven (HMT72G420, Munich, Germany), operating at a frequency of 2.45 GHz with a power output of 360 W. Samples were placed in a microwave-safe borosilicate glass beaker (1000 mL). The sample was positioned centrally on the rotating plate of the microwave with a 24.5 cm diameter ensuring uniform microwave exposure. The total treatment time using each piece of equipment and the conditions are provided in Table 1.

In the initial stage of production, red beetroot and apples were thoroughly washed, peeled, cored, and uniformly cut into 2 cm cubes. They were combined in a 1:1 ratio with an equal amount of water and subjected to 11 different pretreatment conditions (Table 1). After boiling, the softened mixture was pureed using a homogenizer (Arzum, AR1105 Ottimo, İstanbul, Türkiye), and 25% of it was set aside for mixing with tapioca starch and citric acid (additives). For Conditions 1 to 10, the remaining puree was concentrated at 100–103 °C, while the starch-containing puree was gradually added at set soluble solids levels (30 °Bx for 40 °Bx, 15 °Bx for 20 °Bx). The concentration process was completed when the gel-like mixture, known as “herle”, reached the target °Bx value. For Conditions 11 and 12, the additives were added directly to the puree without any further concentration step. The herle was stored at +4 °C for 1–2 days before drying.

After the pretreatments were completed, 25.0 ± 0.5 g of herle was evenly spread onto greaseproof paper using an 8 cm × 8 cm × 0.5 cm mold and a spatula. Drying was carried out in a conventional cabinet-type dryer (Yücebaş Makine Tic. Ltd. Şti., İzmir, Türkiye) at 70 °C, with 20% relative humidity and an air velocity of 2 m/s. The process continued until the pestils’ moisture content reached 0.09–0.10 g water/g dry matter (DM). Once drying was complete, a sufficient amount of pestil was set aside for sensory and color analyses. The remaining samples were ground using a mortar grinder (Retsch RM 200, Haan, Germany) and stored at –20 °C until further analysis.

### 2.3. Chemicals and Solutions

Folin–Ciocalteu reagent, sodium carbonate, gallic acid (purity > 98%), and ethanol were obtained from Merck (Darmstadt, Germany) for the determination of the total phenolic compounds. For total flavonoid contents analysis, sodium nitrite and aluminum chloride were purchased from Merck (Darmstadt, Germany), sodium hydroxide was purchased from Sigma-Aldrich (Steinheim, Germany), and rutin (purity > 98%) was purchased from Acros Organics (Thermo Fisher Scientific, Morris Plains, NJ, USA). Trifluoroacetic acid and acetonitrile were used for polyphenol characterization. All chemicals and solvents used for spectrophotometric and liquid chromatographic analyses were of analytical and chromatographic grades, respectively. The following analytical standards (purity > 98%) were used for the quantification of polyphenols: caffeic acid and vanillin, from HPC Standards (Borsdorf, Germany), chlorogenic acid, ferulic acid, epicatechin, taxifolin, quercetin, and *o*-salicylic acid (TRC, Ontario, Canada), and isoquercitrin and *p*-coumaric acid from Sigma-Aldrich Inc. (St. Louis, MO, USA).

For the hydroxymethylfurfural (HMF) analysis, Carrez I (potasium ferrocyanide, 15% *w*/*v*) and Carrez II (zinc acetate 30% *w*/*v*) solutions (analytical grade), and HMF standard (purity ≥ 99.5%) were sourced from Merck (Darmstadt, Germany).

For elemental analysis, Suprapur-grade 65% nitric acid (HNO_3_) and 30% hydrogen peroxide were obtained from Merck (Germany). Argon and helium gases, each with 99.99% purity, were used. Ultrapure water (ddH_2_O) from the Elga Purelab Option-S 7 system (ELGA LabWater, VWS Ltd., High Wycombe, UK) was used throughout all stages of the analyses.

### 2.4. Sensory Analyses

The red beetroot pestils were evaluated using a rating test for quality parameters and a hedonic scale test for overall preference. A sensory analysis was conducted by a pre-trained panel of 7 individuals, balanced in gender representation and trained according to international standards [39,40,41,42]. Pestil samples cut into 2 cm × 2 cm squares were served in randomized order and labeled with three-digit codes to ensure blinding. Evaluations were conducted in individual sensory booths under controlled environmental conditions (22 ± 1 °C, neutral lighting). Unsalted breadcrumbs and water at room temperature were provided to neutralize palate between samples.

In the hedonic scale test, various attributes of the pestils were evaluated using a nine-point scale (ranging from “9”—“like very much” to “1”—“dislike extremely”). The assessed parameters included color, appearance, overall taste, odor, beetroot-specific aroma, beetroot-specific odor, chewability, texture, and overall acceptability.

Additionally, a rating test (4 = very good, 3 = good, 2 = moderate, 1 = poor) was conducted to evaluate the quality characteristics of pestils in terms of color, appearance, taste, and aroma, based on the Turkish national standards for grape, apricot, plum, and mulberry pestils [35,36,37,38]. The acceptance criterion was set at a minimum score of 3. Sensorial quality parameters are presented in Table 2.

### 2.5. Physicochemical Analyses

The moisture content of the pestil samples was determined using the AOAC method [43] and expressed as a percentage (%). The soluble solids content was measured as °Bx using a digital refractometer (RFM 960, Bellingham + Stanley), following a standard method [44]. The water activity of the samples was measured directly using a LabSwift-a_w_ device (Novasina, Lachen, Switzerland) at 25 °C.

The color of the red beetroot pestil samples was analyzed using a CR-5 Konica Minolta color analyzer (Osaka, Japan). The L* parameter represents lightness (0 = black, 100 = white), a* indicates redness (+) or greenness (−), and b* denotes yellowness (+) or blueness (−). The C* value (chroma or color saturation) ranges from 0 (dull) to 60 (vivid). Additionally, C* and h° values were calculated using Equations (1) and (2). The h° (hue angle) value represents the color tone. The h° values correspond to specific colors, as follows: 0° (red), 90° (yellow), 180° (green), 270° (blue), and 360° (red) [8]. Equations (1) and (2) are as follows:

C* = (a^2^ + b^2^)^1/2^ (1)

h° = tan^−1^(b/a) (2)

Moreover, the high temperatures and extended heating process used in pestil production may lead to browning. Therefore, the browning index (BI) was calculated using the following Equation (3) [45]:

*BI* = [100((*a* + 1.75L*)/(5.645L* + a* − 0.3012b*) − 0.31)] ÷ 0.17 (3)

### 2.6. Hydroxy Methylfurfural (HMF)

**Figure 3:**
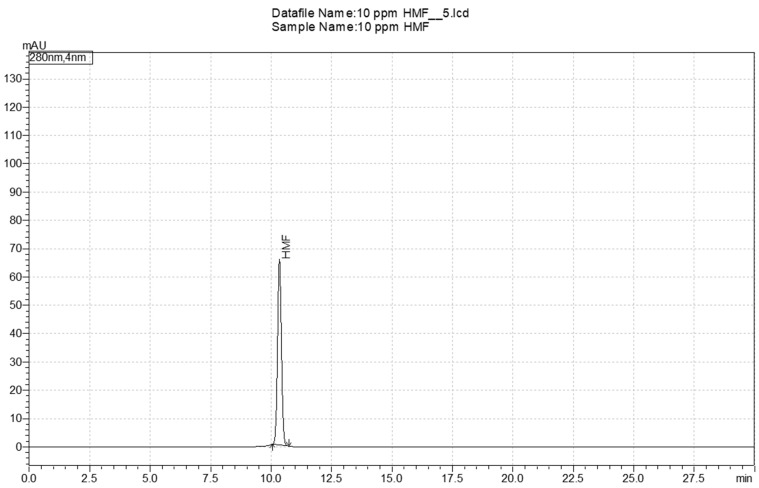
Chromatogram of the HMF standard.

The determination of hydroxymethylfurfural (HMF) in pestil samples was carried out using a modified method based on the work of Rufián-Henares and Delgado-Andrade [46]. In the first step, 7 mL of distilled water was added to 1.00 ± 0.01 g of homogenized pestil sample, which was then vortexed. The mixture was centrifuged at 4500× *g* for 10 min at 4 °C, and the supernatant was transferred to a separate tube. Centrifugation was repeated after the addition of 2 mL of distilled water to the residue, and the resulting supernatants were combined. To the combined supernatants, 0.250 mL of Carrez I solution and 0.250 mL of Carrez II solution were added. The mixture was then centrifuged again under the same conditions, and the final volume was adjusted to 10 mL with distilled water. The solutions were filtered through 0.45 μm PVDF (polyvinylidene fluoride) membrane filters and transferred into 1.2 mL vials for injection into a high-performance liquid chromatography–photodiode array detector (HPLC-PDA, Shimadzu.Co, LC-2030C 3D Plus, Tokyo, Japan). Data acquisition was performed using LabSolution software 5.106 (Shimadzu, Tokyo, Japan). HMF was identified based on its retention time on the column and its characteristic UV spectrum (Figure 3). The amount of HMF was quantified using a calibration curve (R^2^ > 0.999) created from standard solutions with concentrations ranging from 0.5 to 50 mg/kg.

### 2.7. Mineral Composition

To determine the mineral composition of the pestil samples, homogenized samples were digested in a closed-system microwave digestion unit (Milestone Ethos UP, Sorisole, Bergamo, Italy) using 65% HNO_3_ and 30% hydrogen peroxide, following the NMKL 186 method [47]. The digested samples were then diluted to a final volume of 50 mL with ultrapure distilled water and filtered through a 0.45 μm membrane filter. Elemental concentrations were quantified in mg/kg using an inductively coupled plasma–mass spectrometer (ICP-MS) equipped with a Nexion 350 ICP-MS system (PerkinElmer Inc., Shelton, CT, USA). The instrument was operated under the following conditions: *radio frequency power (RF)*: 1600 W, *plasma argon flow rate*: 20.0 L/min, *nebulizer flow rate*: 0.98–1.39 L/min, *auxiliary gas flow rate*: 1.2 L/min, *sample uptake rate*: 20 rpm, *dwell time*: 50 ms, number of replicates: 3, scanning mode: peak hopping, and detector mode: dual. For the quantitative analysis, a linear calibration curve (R^2^ > 0.999) was employed, covering at least five concentration levels, namely 0.05–25 μg/L for microelements and 1–1000 μg/L for macroelements. Calibration standards were prepared by diluting a mixed stock standard solution (PerkinElmer, Inc., Shelton, CT, USA) at a concentration of 10 μg/mL in 5 wt% (*w*/*v*) HNO_3_ using 5 wt% (*w*/*v*) HNO_3_ as the diluent.

### 2.8. Total Betalain Content

Water was added to the homogenized samples at a ratio of 1:15 (*w*/*v*), and the mixture was placed in an ultrasonic bath at 60 °C for 40 min. The resulting extract was then filtered. The aqueous extract of the pestil samples was centrifuged at 1789× *g* rpm for 5 min, and the concentrations of pigments (betacyanin and betaxanthin) were determined by measuring the absorbance at two different wavelengths (483 and 535 nm) using a 1 cm quartz cuvette in a spectrophotometer. The results were calculated using the formula provided in the following Equation (4) [48,49] and expressed as mg of pigment per kg of extract:

*BC* = *A* × *DF* × *MW* × *V_d_* × 1000 × ε × I × *W_d_* (4)

where BC is betalain content (mg/kg), A is the absorbance at the 535 nm (for betacyanin) and 483 nm (for betaxanthin) wavelengths, DF is the dilution factor, MW is the molecular weight of betacyanin (550 g/mol) and betaxanthin (308 g/mol), V_d_ is the volume of extraction solution (mL), **ε** is the molar extinction coefficient, I is the path length of the cuvette (cm), and W_d_ is the sample amount (g)

### 2.9. Total Antioxidant Capacity, Total Phenolic and Flavonoid Contents, Polyphenols

#### 2.9.1. Extraction

Pestil samples were extracted using the method described by Kamiloglu and Capanoglu [50]. 5 mL of extraction solution (an aqueous solution containing 75% methanol and 0.1% formic acid) was added to 2 g of homogenized sample and kept in a cooled ultrasonic water bath (Bandelin Sonorex RK 510 H, Berlin, Germany) for 15 min. The samples were then centrifuged (Hitachi CF15RN, Tokyo, Japan) at 10,000× *g* for 10 min at 4 °C, and the upper liquid phase was collected. This extraction procedure was repeated two more times. The collected supernatants were combined in a single tube and stored at −20 °C until analysis.

#### 2.9.2. Total Antioxidant Capacity

The total antioxidant capacity (TAC) of the samples was measured with two different spectrophotometric assays (using a Shimadzu UV-1700 spectrophotometer, Tokyo, Japan).

For the CUPRAC analysis, the procedure described by Apak et al. [51] was followed. Cuvettes were loaded with 100 μL of the extracted sample, followed by the sequential addition of 1 mL of copper (II) chloride, 1 mL of neocuproine, 1 mL of ammonium acetate, and 1 mL of double-distilled water (ddH_2_O). After incubating the mixture in the dark for 30 min, its absorbance was measured at 450 nm. The results were expressed as mg Trolox^®^ equivalent (TE) per 100 g of sample (100–1000 ppm, R^2^ ≥ 0.998).

For the DPPH analysis, the procedure described by Kumaran and Karunakaran [52] was followed. Briefly, 100 μL of the sample was added to cuvettes, followed by the addition of 2 mL of DPPH solution. After incubating the mixture in the dark for 30 min, its absorbance was measured at 517 nm. The results were expressed as mg Trolox^®^ equivalent (TE) per 100 g of sample (10–200 ppm, R^2^ ≥ 0.993).

#### 2.9.3. Total Phenolic Compounds

Total phenolic content was determined using the Folin–Ciocalteu reagent, following the method described by Velioglu et al. [53]. In summary, 100 μL of extracted sample was added to cuvettes, followed by the addition of 750 μL of Folin–Ciocalteu reagent. The mixture was then incubated for 5 min, after which 750 μL of a 6% sodium carbonate solution was added. Following a 90 min incubation, the absorbance of the mixture was measured at 725 nm using a spectrophotometer (Agilent Cary 60, Santa Clara, CA, USA). The results were expressed as mg gallic acid equivalent (GAE) per 100 g of sample and calculated using a calibration curve (10–800 ppm, R^2^ ≥ 0.999).

#### 2.9.4. Total Flavonoid Compounds

The total flavonoid content of red beetroot was determined using the method described by Kim et al. [54]. A 1 mL aliquot of the extracted sample was added to cuvettes, followed by the addition of 300 μL of 5% sodium nitrite solution. The mixture was incubated for 5 min, after which 300 μL of 10% aluminum chloride solution was added and incubated for an additional 1 min. Following incubation, 2 mL of 1 M NaOH and 2.4 mL of double-distilled water (ddH_2_O) were added to the mixture. The absorbance was then measured at 510 nm, and the results were expressed as mg rutin equivalent (RE) per 100 g of sample (10–800 ppm, R^2^ ≥ 0.999).

#### 2.9.5. Polyphenol Composition

The identification and quantification of phenolic compounds were carried out using liquid chromatography–tandem mass spectrometry (LC-MS/MS 8060 Shimadzu. Co 8060, Kyoto, Japan). A C18 column (3 × 100 mm, 3 μm, GL Sciences, Tokyo, Japan) was used for chromatographic separation. The extracted samples were filtered through 0.22 μm PVDF (polyvinylidene fluoride) membrane filters (and transferred into LC-MS/MS vials) as previously described in the literature [55]. Then, 10 μL of each filtered sample were injected into the system, using formic acid/ultrapure water (1:1000, *v*/*v*; eluent A) and formic acid/acetonitrile (1:1000, *v*/*v*; eluent B) as the mobile phases, at a flow rate of 0.4 mL/min. The gradient elution program was as follows: 0 min, 20% B; 0.0–0.5 min, 20% B (isocratic); 0.5–7.0 min, 20–50% B (linear gradient); 7.0–12.0 min, 50–95% B (linear gradient); 12.0–12.1 min, 95–20% B (linear gradient); and 12.1–15.0 min, 20% B (isocratic). The column temperature was maintained at 40 °C. The mass spectrometer operated in multiple reaction monitoring (MRM) mode with electrospray ionization (ESI) in both positive and negative modes. Instrument parameters were set as follows: a nebulization gas (N_2_) flow rate of 3.0 L/min, a drying gas (N_2_) flow rate of 10.0 L/min, an interface voltage of 4.0 kV, a desolvation line temperature of 250 °C, an interface temperature of 300 °C, and a heat block temperature of 400 °C. Data acquisition was performed using LabSolution software (Shimadzu). Phenolic compounds were identified by comparing retention times, as well as the target and daughter ion transitions of sample peaks with those of calibration standards. Concentrations were calculated using a linear calibration curve (R^2^ > 0.999) prepared from standard solutions at concentrations ranging from 0.5 to 50 µg/kg.

### 2.10. Statistical Analyses

Pestil production under each pretreatment condition (Table 1) was carried out in triplicate (*n* = 3) as biological replicates to ensure reproducibility. Physicochemical, spectrophotometric, and chromatographic analyses were performed in triplicate as technical replicates (*n* = 3). Results for each pestil sample are expressed as mean ± standard deviation.

The experimental design involved the independent production of pestils under 11 distinct pretreatment conditions, each representing a unique combination of boiling method and concentration parameters, including target °Brix values. These pretreatment conditions were deliberately selected and treated as fixed levels of a single factor. A standardized drying protocol was applied across all trials to maintain consistency. To compare the effects of the different pretreatment conditions on the quality of the pestil, a one-way ANOVA was used to analyze the differences between group means. ANOVA is a parametric test that determines whether there are statistically significant differences among the means of three or more independent groups.

The data were analyzed using one-way analysis of variance (ANOVA) with SPSS 28.0 software, and Tukey’s test was applied to identify significant differences between samples (*p* < 0.05). Moreover, Pearson correlation coefficients were calculated to explore the relationships between variables. Principal component analysis (PCA) was used to identify patterns and highlight similarities and differences among red beetroot pestil samples. Hierarchical cluster analysis (HCA) was used as a statistical technique to group objects into clusters based on their similarity or distance. PCA and HCA were performed using Minitab 21.0 software.

## 3. Results and Discussion

### 3.1. Variations in Total Heat Treatment Period

**Figure 4:**
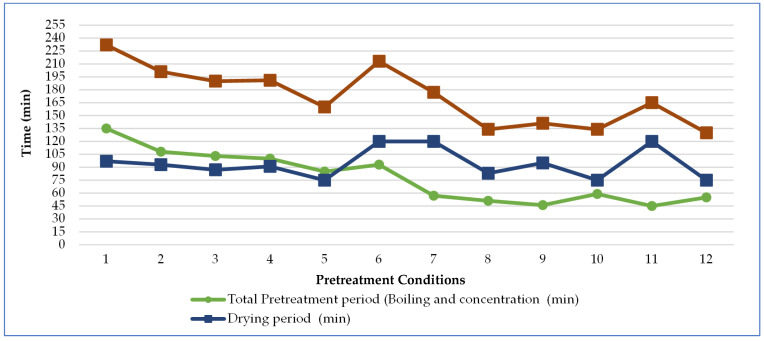
Variations in pretreatment, drying, and total thermal processing times.

This study investigated the effects of various pretreatment conditions on the quality of red beetroot pestil, using a constant drying process. The drying kinetics of the final product were discussed previously in our recent publication [56]. Pretreatments included traditional boiling, thermosonication, and microwave-assisted thermosonication, with concentration carried out up to 40 or 20 °Bx (Conditions 1–10). In Conditions 11 and 12, pestil was produced using drained and pureed beetroot pulp without undergoing the further concentration step. Figure 4 illustrates the variations in the durations of the pretreatment, drying, and total thermal processing stages. The longest pretreatment time (135 min) occurred with traditional boiling (Condition 1), while the shortest (45 min) was recorded for Condition 11. The drying time ranged from 75 to 120 min as the soluble solid content decreased. The total thermal processing time was longest in Condition 1 (232 min) and shortest in Condition 12 (130 min).

Overall, thermosonication and microwave-assisted boiling (Conditions 2–4 and 6–10) reduced pretreatment and total process times by 13–42% and 20–51%, respectively, compared to traditional boiling (Control, Condition 1). Red beetroot pestils produced without concentration (Conditions 11 and 12) showed time reductions of 23–43% and 59–67%, respectively.

### 3.2. Sensorial Quality

The sensory evaluation of red beetroot pestil samples was conducted using a hedonic scale test and a scoring test [35,36,37,38]. In the hedonic test, attributes, such as color, appearance, taste, odor, beetroot-specific aroma, texture, and overall acceptability, were assessed on a nine-point scale (Table 3). The sensory analysis of twelve beetroot-based formulations, including two traditional controls (Condition 1 and 6), revealed statistically significant differences (*p* < 0.05) across multiple sensory attributes. Condition 11 demonstrated significantly higher mean scores in key attributes, such as color (8.1 ± 1.1), taste (7.4 ± 0.7), texture (7.5 ± 0.9), and overall acceptability (7.5 ± 0.9), compared to several other samples, indicating a marked preference among panelists. Similarly, Conditions 4 and 7 also achieved high acceptability scores, with statistically comparable scores to Condition 11. In contrast, Condition 12 exhibited significantly lower scores (*p* < 0.05) for taste (5.2 ± 0.9), odor (beetroot-specific aroma (5.2 ± 0.9), and chewability (5.0 ± 0.8), suggesting limited sensory appeal. Overall, with mean scores ranging from 5.9 ± 0.6 to 7.5 ± 0.4, the beetroot pestil samples produced using various pretreatment methods were generally well accepted by the panelists. These findings suggest that innovative pretreatments can significantly improve the sensory quality of beetroot-based snacks compared to traditional formulations.

Pestil-specific attributes were also assessed using a four-point scoring test, with a minimum score of 3 required for acceptability according to product standards [35,36,37,38]. All samples met this requirement, and the highest score (3.9 ± 0.4) was recorded for Condition 10, demonstrating superior sensory quality.

### 3.3. Physicochemical Properties

The moisture content of red beetroot pestil samples ranged from 8.76% to 10.23%, with water activity between 0.60 and 0.67. According to pestil product standards [35,36,37,38], the maximum allowable moisture content is 15–20%, depending on the fruit type. Drying was performed until the moisture level fell below this threshold, ensuring compliance with product standards. Research indicates that maintaining moisture below 15% prevents microbial growth and undesirable changes, like sugar crystallization, browning, flavor loss, and lipid oxidation [57]. Factors, such as drying time, temperature, and thickness, significantly affect moisture content. Studies on pomegranate, date, and mulberry pestils have shown moisture content variations similar to those observed in red beetroot pestil, further supporting the drying conditions used in this study [8,58].

The color values of red beetroot pestil samples are presented in Table 4. Color is a key quality attribute influencing consumer acceptance, and the results indicated that different pretreatment methods had a significant effect on color parameters (*p* < 0.05). The color of red beetroot pestil can be described as brownish–red. Condition 9 showed the most intense red and saturated appearance, with the highest a* value (14.41 ± 0.11) and chroma (C*) value (15.07 ± 0.10), along with a slightly yellowish–red tone due to its higher b* value. Conditions 11 and 12 also had vivid red and saturated colors, supported by their high a* and C* values. Condition 8 had a high a* value (10.77 ± 0.28), reflecting a strong red hue, but its lower b* value gave it a cooler, less vibrant tone. Traditional Control 1 (Condition 1) had the highest L* value (30.80), indicating the brightest and lightest sample, with a balanced reddish-yellow color. In contrast, Conditions 2, 3, and 4 showed low b* and C* values, resulting in dull and pale colors. Traditional Control 2 (Condition 6) had the lowest a*, b*, and C* values, making it the least saturated and faded in appearance. Conditions 5 and 7, with lower L* values, appeared darker with moderately red tones.

Studies by Şengül and Ünver [11] and Özaltın and Çağındı [59] reported variations in the color parameters of cranberry and grape pestils, respectively. These differences in L*, a*, b*, C*, and h° values can be attributed to variations in fruit type, formulation, and processing conditions. The browning index (BI) in pestil production is influenced by high temperatures and prolonged heat treatment. Red beetroot pestils exhibited relatively low BI values (12.39–39.75), likely due to their brownish-red color. In contrast, dried red beetroot powder has higher BI values (76.28–105.91), primarily due to differences in drying methods and cultivars [60]. Moreover, heat treatment, particularly boiling, inactivates polyphenol oxidase, contributing to the lower BI in pestils. The literature shows that BI values vary based on raw materials and processing methods. For example, pear pomace has a high BI (151.03), while apple and quince leathers have values of around 80. In contrast, maqui extract lowers the BI to 4–10, likely due to its dark purple color and antioxidant properties. [61,62].

### 3.4. Hydroxymethyl Furfural (HMF) Content of the Pestils

The levels of HMF detected in beetroot pestils are presented in Table 4. HMF content in red beetroot pestils was highest (282.01 ± 5.85 mg/kg) under traditional production conditions (Condition 1) but decreased to between the LOQ and 2.23 mg/kg in Conditions 6 to 12. These results comply with the criteria set by the Turkish Standards Institute, which specifies a limit of 50 mg/kg [35,36,37,38]. Although there are no specific studies on HMF levels in beetroot pestils, several studies have investigated HMF content in other types of pestils and molasses. For example, HMF levels have been reported as 231.76 mg/kg in beet molasses [63], 67.92–140.42 mg/kg in mulberry pestil [10], and 7.89–333.33 mg/kg in pomegranate pestil [8]. Additionally, drying techniques have a significant impact on HMF levels, with apricot pestils showing 45.64 mg/kg for sun drying, 19.39 mg/kg for vacuum drying, and 13.62 mg/kg for microwave drying [64].

The present study demonstrated that hybrid pretreatments involving thermosonication and microwave significantly reduced HMF formation in beetroot pestils, particularly under Conditions 6–12. The reduction in HMF levels is attributed to the shorter exposure to high temperatures during the boiling (95 °C) and concentration (103 °C) steps, or their complete omission in certain conditions. Reducing the soluble solids content from 40 °Bx to 20 °Bx before drying under Conditions 6–12 significantly shortened the concentration step, leading to the most substantial reductions. Although this led to a longer drying period, the overall decrease in high-temperature exposure had a more pronounced effect on minimizing HMF formation. These process optimizations reduced the HMF content in the final product to levels below the limit of quantification (LOQ). Consistent with our findings, reports in the literature indicate that HMF concentrations are significantly influenced by various factors, such as product type, sugar addition, presence or absence of oxygen, magnetic waves, production times, cooking conditions, drying methods, etc. [9,15,58,64,65,66].

However, these findings contrast with several reports in the literature suggesting that microwave treatment may promote HMF formation [8,9,15,66]. This discrepancy likely stems from differences in microwave power, exposure time, moisture content, and food matrix composition. Microwave heating can accelerate the Maillard reaction and caramelization processes, particularly under low-moisture and high-temperature conditions, resulting in increased HMF production. In dry systems or during prolonged high-power treatments, localized overheating may occur, leading to elevated levels of HMF and other undesirable thermal degradation products. Conversely, in systems, like that of the present study, where microwave pretreatment was applied under moist conditions and for brief durations, the risk of HMF formation appears to be mitigated. By reducing the need for extended high-temperature processing, hybrid approaches that include microwave treatment can effectively lower the thermal load and suppress HMF generation [67,68]. These results align with research indicating that microwave-assisted drying or blanching under optimized parameters can result in lower HMF levels compared to conventional thermal methods [67,68].

Therefore, the effect of microwave treatment on HMF formation is highly context-dependent. Careful optimization and control of processing parameters are critical for maximizing the benefits of microwave technology while minimizing the formation of undesirable compounds. Overall, these findings underscore the substantial impact of processing parameters and product type on the formation of HMF in food products.

### 3.5. Mineral Composition

The mineral content of red beetroot pestil samples are shown in Table 5. This study revealed that the red beetroot pestil samples are particularly rich in essential macro-elements, including potassium (K), sodium (Na), phosphorus (P), and magnesium (Mg), reflecting the natural composition of the raw beetroot material [15]. Trace elements, such as zinc (Zn), iron (Fe), and manganese (Mn), were also detected and quantified using the employed analytical method.

These minerals play crucial physiological roles. Phosphorus is essential for protein synthesis, enzyme activity, water balance, and digestive secretions [69]. Calcium (Ca) contributes to bone and tooth strength and is vital for cellular structural stability, while magnesium (Mg) is necessary for enzymatic activity. Iron (Fe) supports electron transport, enzyme activation, and immune function [70]. These findings suggest that red beetroot pestil is not only nutritionally valuable but may offer functional health benefits as a healthy snack alternative. Furthermore, the mineral content measured in this study is higher than that reported in other types of pestils in the literature [13,15,71,72,73].

The data in Table 5 also reveal that different pretreatment conditions significantly influenced the mineral content of the pestils. In particular, Condition 2 resulted in the highest mineral concentrations, while Condition 8 exhibited the lowest. Although the effects of thermosonication, ultrasonication, and microwave pretreatments on pestils have not been widely reported, similar studies on various food products indicate that such treatments can either enhance or reduce mineral levels. These effects are often attributed to various mechanisms, like cavitation, cellular disruption, and changes in mineral solubility. For instance, ultrasound treatments can promote homogenization and enhance the release of intracellular minerals into the food matrix or surrounding medium [74].

The observed inconsistencies in mineral retention, particularly fluctuations in calcium (Ca) and potassium (K) levels across different pretreatment conditions, can be attributed to several processing-related factors. Thermal treatments, such as boiling, often lead to the leaching of water-soluble minerals into the cooking medium, thereby reducing their concentrations in the final product. Additionally, certain techniques, like ultrasonication and microwave processing, can disrupt cellular structures, enhancing the release of intracellular minerals. However, this disruption may also increase the susceptibility of minerals to leaching during subsequent processing steps. Variations in processing parameters, including temperature, duration, and intensity, further influence mineral stability and solubility, contributing to the inconsistent trends identified in this study [75,76].

While this study measured the total mineral content of red beetroot pestil, it did not assess mineral bioaccessibility—the proportion of minerals available for absorption in the human gastrointestinal tract. Processing methods can significantly impact bioaccessibility by altering the food matrix or affecting the presence of antinutritional factors that bind minerals. To comprehensively evaluate the nutritional implications of different processing techniques, future research should employ standardized in vitro digestion models, such as the INFOGEST protocol, which simulates human gastrointestinal conditions and allows for the assessment of nutrient bioaccessibility [77,78].

### 3.6. Total Betalain Content

The betaxanthin, betacyanin, and total betalain concentrations in the red beetroot pestils are presented in Table 6.

The highest concentrations of betaxanthin, betacyanin, and total betalains were observed under Condition 4 (257.10 ± 18.52 mg/kg, 355.90 ± 13.22 mg/kg, and 613.00 ± 31.70 mg/kg, respectively), while the lowest levels were obtained under Condition 2 (128.68 ± 13.02 mg/kg, 171.64 ± 15.69 mg/kg, and 300.31 ± 28.71 mg/kg, respectively). The enhancement under Condition 4 can be attributed to the effects of microwave-assisted processing.

Microwave treatments are known to facilitate rapid and uniform heating, promoting cell wall disruption and enhancing the release of intracellular compounds, such as betalains. Furthermore, the short processing durations involved help minimize exposure to high temperatures, thereby reducing the risk of thermal degradation of sensitive bioactive compounds [79]. These characteristics explain the improved betalain retention observed under microwave-assisted conditions.

The thermal degradation kinetics of betalains—specifically betacyanins and betaxanthins—have been studied in various contexts. Betacyanins are generally more stable than betaxanthins, with both classes showing degradation patterns influenced by temperature, pH, and oxygen exposure. For instance, betacyanin degradation typically follows first-order kinetics, with higher temperatures accelerating pigment loss. Betaxanthins, in contrast, are more heat-labile and degrade more readily under thermal processing [80,81,82].

Although no previous studies have evaluated betalain stability specifically in red beetroot pestil, research on drying and extraction methods in red beetroot indicates that microwave-assisted extraction enhances betalain recovery while preserving pigment integrity. These improvements are attributed to reduced thermal exposure and efficient cell matrix disruption [79,83].

Consequently, this study supports the conclusion that processing conditions, particularly microwave-assisted methods, have a significant impact on betalain retention in red beetroot pestil. These findings emphasize the importance of optimizing thermal parameters to preserve the nutritional and functional quality of the final product.

### 3.7. Total Antioxidant Capacity (TAC), Total Phenolic Compound (TPC) and Total Flavonoid Compound (TFC) Values

The TAC, TPC, and TFC results are presented in Table 7.

The total antioxidant capacity (TAC) of red beetroot pestils was determined using two assays, namely the CUPRAC and DPPH methods. The CUPRAC method yielded the highest TAC values (182.07 ± 26.48–870.78 ± 25.05 mg TE/100 g DM), followed by the DPPH method (14.05 ± 0.46–48.18 ± 0.76 mg TE/100 g DM). Previous studies have shown that antioxidant capacity results can differ depending on the analytical method applied. The CUPRAC method measures both hydrophilic and lipophilic antioxidants, whereas the DPPH method detects only lipophilic antioxidants. Therefore, the variations in TAC values between the two methods are attributed to their differing mechanisms [15,84,85]. Research has shown that fresh beetroot exhibits a higher antioxidant capacity compared to dried beetroot and puree [86].

Total phenolic content (TPC) and total flavonoid content (TFC) were highest in Condition 6 (48.18 ± 0.76 mg GAE/100 g DM and 313.17 ± 10.63 mg RE/100 g DM, respectively), while the lowest values were obtained in Condition 4 (76.26 ± 2.37 mg GAE/100 g DM and 128.19 ± 3.86 mg RE/100 g DM, respectively).

This study investigated the effects of different pretreatment methods on the TAC of red beetroot pestils and found that Condition 6 (Traditional Control 2) produced the highest TAC. Thermosonication and microwave treatments, used as alternatives, resulted in a reduction in TAC by 9–69% (CUPRAC) and 8–54% (DPPH) compared to the traditional control (Condition 1). These findings indicate that while certain processing conditions enhance antioxidant capacity, others may have a detrimental effect.

Similarly, thermosonication and microwave applications led to a reduction in TPC and TFC. Compared to Condition 1, thermosonication resulted in a 1–62% decrease in TPC and 0.5–64% decrease in TFC of red beetroot pestils.

To date, no published studies have investigated the use of ultrasonic or microwave treatments as pretreatments in pestil production. However, several studies have reported that thermal and ultrasonic processes may have varying effects on the TPC and TAC in food systems [87]. While certain studies suggest that ultrasonic and microwave treatments enhance antioxidant activity in certain plant products [88,89], others report a decrease in antioxidant content. Additionally, some drying methods, such as microwave–hot air drying and ultrasonic pretreatment, significantly affect phenolic content [27,89]. Some drying methods are effective in preserving flavonoids, while traditional drying techniques often results in flavonoid degradation [86,90].

Overall, our findings align with the existing literature and demonstrate that pretreatment methods significantly (*p* < 0.05) affect the TAC, TPC, and TFC of red beetroot pestils. These results highlight the importance of optimizing processing conditions to improve the nutritional quality of the final product.

### 3.8. Characterization of Polyphenols by UPLC-ESI-MS/MS

**Figure 5:**
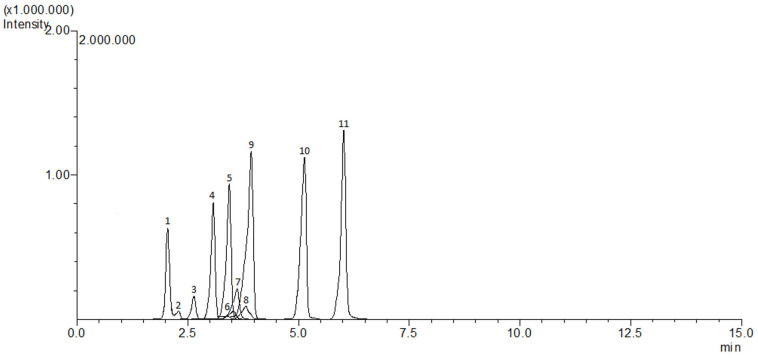
Chromatogram of polyphenols. (1) Chlorogenic acid (2.01 min), (2) epicatechin (2.43 min), (3) caffeic acid (2.67 min), (4) rutin (3.10 min), (5) isoquercitrin (3.45 min), (6) p-coumaric acid (3.54 min), (7) vanillin (3.55 min), (8) ferulic acid (3.79 min), (9) taxifolin (3.87 min), (10) o-salicylic acid (5.06 min), and (11) quercetin (5.95 min).

UPLC-ESI-MS/MS analysis of red beetroot pestils revealed 11 major compounds, including 5 flavonoids, 6 phenolic acids, and other bioactive substances (Table 8 and Figure 5). The majority of the compounds were identified in negative ion mode. The identified phenolics are consistent with the studies reported in the literature [91,92,93,94,95,96].

#### 3.8.1. Flavonoids

Five flavonoids were identified in red beetroot pestils, with epicatechin being the predominant compound, accounting for an average of 93.76% of the total flavonoid content, respectively (Table 9). Flavonol derivatives are valuable phytochemicals known for their antioxidant and anticarcinogenic properties [92].

The flavonoid composition of red beetroot pestils varied significantly (*p* < 0.05). Epicatechin was the most abundant flavonoid, with the highest concentration in Condition 7 and the lowest in Condition 4. Isoquercitrin followed, peaking in Condition 2 and reaching its lowest concentration in Condition 4. Rutin was most abundant in Condition 11 and least abundant in Condition 4. Quercetin had its highest level in Condition 3 and its lowest in Condition 10. Lastly, taxifolin peaked in Condition 7 and was lowest in Condition 5. Overall, red beetroot pestils showed a higher flavonoid yield, particularly for epicatechin and isoquercitrin. These results align with the findings of Borjan et al. [92], who identified epicatechin as one of the most abundant flavonoids in all beetroot extracts. Similarly, Tumbas Šaponjac et al. [91] reported high levels of epicatechin and catechin. In their study, the concentrations of epicatechin, catechin, and quercetin in beet pulp extracts were reported as 3.88 g/100 g, 156.04 g/100 g, and 0.41 g/100 g, respectively.

When the effects of pretreatment conditions were compared to the traditional processing conditions (Traditional Control—Condition 1), an increase of 4–59% was observed for epicatechin, 10–16% for rutin, 7–35% for isoquercitrin, 14–184% for quercetin, and 1–67% for taxifolin. However, the concentration of epicatechin was reduced by 11–75% relative to the control. Similarly, the levels of rutin, isoquercitrin, quercetin, and taxifolin decreased by 1–46%, 11–52%, 36–63%, and 8–31%, respectively. Overall, these results indicate significant differences (*p* < 0.05) in flavonoid content across different pretreatment methods. It was also noted that the effects varied depending on the specific pretreatment applied and the chemical structure of the flavonoids. For instance, extending the thermosonication process from 30 to 45 min led to a 1–36% increase in taxifolin levels in the pestils, while the rutin content decreased by 12–25%.

Among the identified flavonoids, the highest levels of rutin, taxifolin, and quercetin were found in pestils produced under Conditions 3, 7, and 11, respectively. Borjan et al. [92] also reported high flavonoid levels using ultrasonic extraction. Similarly, in the present study, the flavonoid content was found to be elevated in ultrasonically pretreated pestils.

#### 3.8.2. Phenolic Acids and Other Polyphenols

Six phenolic acids and other polyphenols were identified in red beetroot pestils, including caffeic acid, *p*-coumaric acid, chlorogenic acid, ferulic acid, *o*-salicylic acid, and vanillin. Chlorogenic acid was the predominant compound, accounting for 0.01% to 98.60% of the total phenolic acid content (Table 10). The levels of phenolic acids and other polyphenols in red beetroot pulps showed a statistically significant difference (*p* < 0.05). Condition 7 had the highest levels of chlorogenic and *p*-coumaric acids, while Condition 4 had the lowest concentrations of chlorogenic acid, ferulic acid, and vanillin. Ferulic acid was most abundant in Condition 1, whereas *p*-coumaric acid was at its lowest in Condition 9. Vanillin and caffeic acid reached their highest concentrations in Condition 2, with caffeic acid being least abundant in Condition 10. Lastly, *o*-salicylic acid peaked in Condition 3 and was at its lowest in Condition 12. According to Borjan et al. [92], protocatechuic acid was the most abundant phenolic acid in all beetroot extracts, followed by chlorogenic acid, caffeic acid, and coumaric acid.

Compared to the traditional pretreatment method (Condition 1), alternative pretreatment conditions resulted in increases in caffeic acid (4–175%), *o*-salicylic acid (0.8–31%), vanillin (32–135%), *p*-coumaric acid (13–45%), and ferulic acid (22–66%), with chlorogenic acid showing a 64% increase in Condition 6. However, the levels of caffeic acid, o-salicylic acid, vanillin, p-coumaric acid, ferulic acid, and chlorogenic acid decreased by 31–45%, 9–51%, 4–38%, 18–51%, 5–50%, and 5–70%, respectively. Overall, significant differences (*p* < 0.05) were detected in phenolic acid and bioactive compound levels across different pretreatment conditions.

Additionally, another study on beet pulp extracts identified protocatechuic acid, gallic acid, chlorogenic acid, ferulic acid, caffeic acid, and coumaric acid [91]. In the study conducted by Gong et al. [95], caffeic acid, ferulic acid, gentisic acid, vanillin, and vanillic acid were identified among the polyphenols present in red beetroot. Furthermore, another study reported the significant presence of gallic acid, chlorogenic acid, caffeic acid, and *p*-coumaric acid [96]. Similarly, Vulic et al. [97] confirmed the presence of protocatechuic, caffeic, and ferulic acids in four beetroot varieties, with phenolic content in beet pulps ranging from 1.87 mg/g to 11.98 mg/g.

### 3.9. Chemometric Analysis

#### 3.9.1. Correlation Coefficient

**Figure 6:**
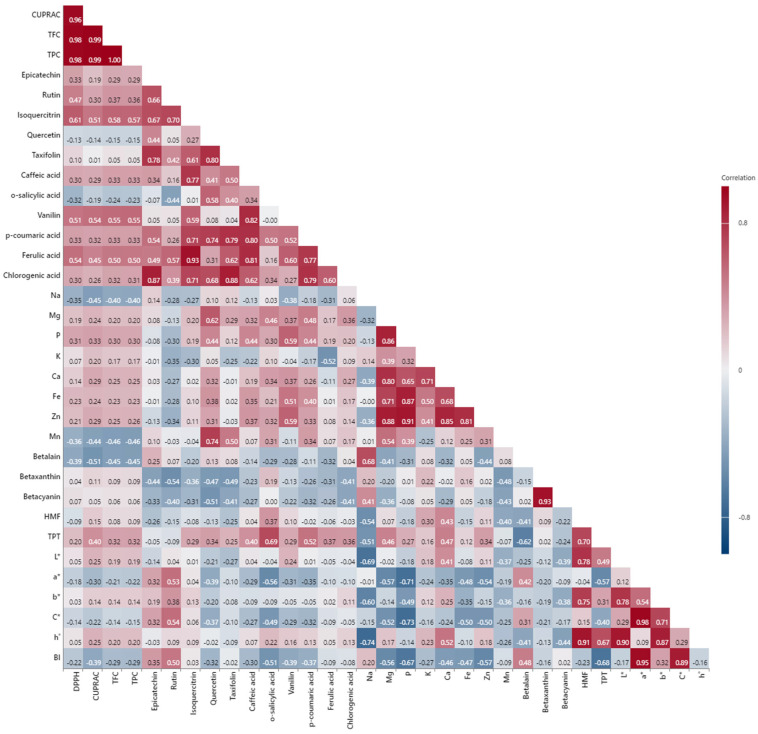
Pearson correlation among chemical components in red beetroot pestils.

To highlight the role of the phenolic content in determining the antioxidant capacity of red beetroot pestil, Pearson correlation analysis was conducted between TAC (measured by two different methods) and TPC. The correlation coefficients between individual phenolic compounds, TPC, and TAC are presented in Figure 6. A strong correlation was found between TPC and TAC assessed by both the DPPH and CUPRAC methods (0.98 ≤ r ≤ 0.99), consistent with findings by Straus et al. [98] and Sawicki et al. [99].

Phenolic compounds exhibit antioxidant properties and may play a role in disease prevention. In this regard, TPC is a key determinant of the antioxidant capacity of red beet pulps subjected to different pretreatments, regardless of the analytical method used [100]. Similarly, TFC showed a strong correlation with antioxidant activity measured by the DPPH and CUPRAC methods (0.98 ≤ r ≤ 0.99). The variation in correlation coefficients across different antioxidant assays suggests that no single test can comprehensively evaluate overall antioxidant capacity. Therefore, multiple analytical approaches using different mechanisms are recommended for a more accurate interpretation of results [101].

Furthermore, previous research has indicated that the Folin–Ciocalteu method may not be entirely specific for TPC analysis, as certain reducing agents can interfere with the results, potentially leading to an overestimation of phenolic concentrations [85]. In line with other studies, a positive correlation was observed between TPC and TAC.

Moreover, the correlation between individual phenolic compounds and antioxidant capacity in red beetroot pestils was investigated (Figure 6). Although no single phenolic compound emerged as the primary contributor, isoquercitrin, vanillin, and ferulic acid exhibited a moderate correlation with TAC in the DPPH assay (0.51 ≤ R^2^ ≤ 0.61). On the other hand, epicatechin, rutin, caffeic acid, p-coumaric acid, and chlorogenic acid showed weak correlations (0.30 ≤ R^2^ ≤ 0.47). Quercetin and o-salycylic acid displayed a low negative correlation (0.13 ≤ R^2^ ≤ 0.32). These weak to moderate correlations suggest that the antioxidant activity of red beetroot pestils likely results from synergistic effects rather from than the influence of individual compounds alone [102].

Similar trends were observed in the CUPRAC assay, with isoquercitrin and vanillin showing moderate correlations (0.51 ≤ R^2^ ≤ 0.54), while other phenolics exhibited weaker associations (0.26 ≤ R^2^ ≤ 0.45). Given the vast diversity of flavonoids (>4000 species) [103], researchers need to conduct further studies to better understand the structure–activity relationships in red beetroot pestils The CUPRAC assay, which measures the total reducing power of antioxidants, often reveals different antioxidant profiles compared to DPPH, as it is influenced by factors, such as pH and the structural characteristics of phenolic compounds [84]. The weak correlations observed for certain phenolics, such as rutin and chlorogenic acid, may be attributed to variations in their redox potential or stability under different assay conditions [104]. Given the complexity of flavonoid interactions, further research is needed to clarify the structure–activity relationships affecting antioxidant capacity in red beetroot pestils.

Mineral element correlations revealed a positive association between quercetin and Mn/Mg (0.62 ≤ R^2^ ≤ 0.74) and between vanillin and Zn/Fe/P (0.51 ≤ R^2^ ≤ 0.59), along with strong inter-element correlations (0.65 ≤ R^2^ ≤ 0.91). Similar findings have been reported, where flavonoids, such as quercetin, form complexes with metal ions—known as metal chelating— enhancing their stability and antioxidant properties [105]. These strong inter-element correlations indicate that red beetroot is a rich source of essential minerals, which may contribute to its health benefits [106]. Additionally, betaxanthin and betacyanin displayed a strong positive correlation (R^2^ = 0.93).

Processing parameters significantly influenced color and chemical composition. HMF showed a strong positive correlation with TPT (total processing time) (R^2^ = 0.70) and color attributes (L*, b*, and h°: 0.75 ≤ R^2^ ≤ 0.91). Total Processing time (TPT) also showed a moderate positive correlation with h° (R^2^ = 0.67) and a moderate negative correlation with BI (R^2^ = −0.68). Significant correlations appeared between the color parameters, as follows: a* showed strong correlations with both C* and BI (0.95 ≤ R^2^ ≤ 0.98), and b* was correlated with C* and h° (0.71 ≤ R^2^ ≤ 0.87). These findings demonstrate that processing conditions significantly influence both the chemical composition and color attributes of red beetroot pestils. The strong positive correlation between HMF and total processing time suggests that prolonged heat exposure promotes Maillard reactions, leading to HMF accumulation [107]. HMF is widely recognized as an indicator of thermal degradation in food products, and excessive formation can negatively impact both nutritional value and safety [108]. The observed correlations between HMF, L*, b*, and h° values indicate that browning reactions occur alongside HMF formation, further altering the visual and sensory properties of beetroot products.

#### 3.9.2. Principal Component Analysis

Principal component analysis (PCA) was used to reduce the dimensionality of large datasets and improve the interpretability of the results. The number of components retained in statistical analysis depends on the dataset and the proportion of variance explained [109].

**Figure 7:**
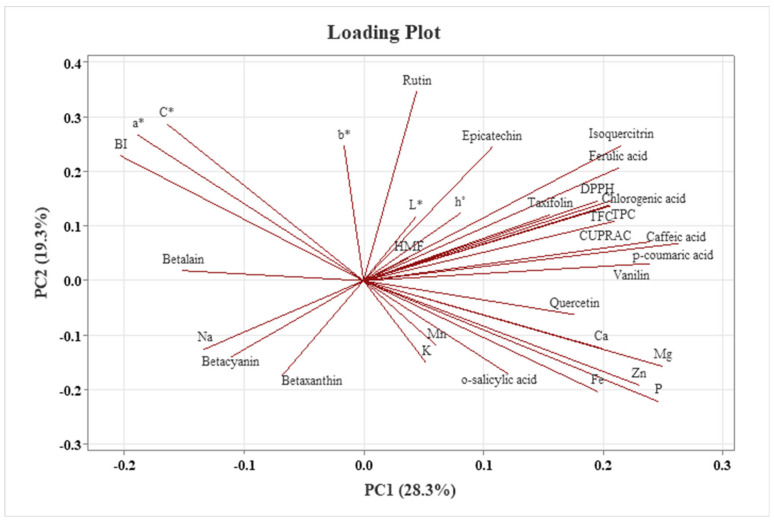
Principal component analysis (PCA) loading plot of red beetroot pestils.

**Figure 8:**
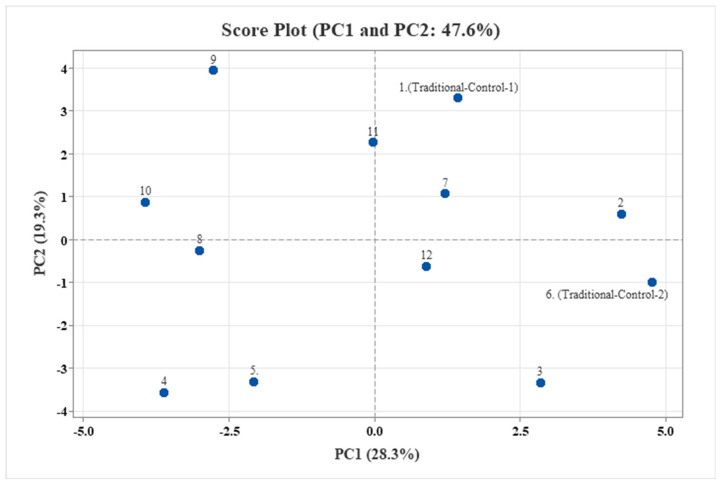
Principal component analysis (PCA) score plot of red beetroot pestils.

Figure 7 presents the loading plot, which illustrates the contribution of each variable—including TAC assays, individual phenolic compounds, elements, HMF, color parameters, and betalain composition—to the principal component scores. Meanwhile, the score plot in Figure 8 represents the distribution of the analyzed samples. In PCA, only components with eigenvalues exceeding 1.00 were retained to ensure analytical reliability. The results indicate that the first seven principal components accounted for 28.30%, 19.30%, 17.20%, 12.80%, 6.90%, 5.80%, and 4.10% of the variance, respectively, yielding a cumulative variance ratio of 94.30% (Table 11).

According to the data presented in Table 11, the positive and negative contributions of the variables to PC1 and PC2 facilitate the identification of key sources of variation in TAC assays, individual phenolic compounds, elements, HMF, color, and betalain composition. PC1 accounts for 28.30% of the total variance, with the highest positive loadings observed for individual phenolic compounds, including *p*-coumaric acid (0.802), caffeic acid (0.729), and vanillin (0.729), as well as elements, such as magnesium (0.764), phosphorus (0.752), and zinc (0.704). These results indicate that these variables significantly contribute to the variance along the PC1 axis and are critical in distinguishing the analyzed samples. Conversely, the analysis showed negative loadings for a* (−0.577), C* (−0.502), BI (−0.619), and betalain (−0.463), indicating an inverse relationship with PC1.

PC2 accounts for 19.30% of the total variance, with significant positive loadings for rutin (0.875), epicatechin (0.616), a* (0.674), C* (0.725), and b* (0.623), highlighting a strong association between PC2 and both phenolic compounds and color parameters. Conversely, Fe (−0.512), P (−0.558), Zn (−0.483), and betaxanthin (−0.436) displayed negative loadings, indicating an inverse relationship with PC2. Together, PC1 and PC2 explain 47.60% of the total variance and serve as the primary contributors to variation in TAC assays, individual phenolic compounds, elements, color, and betalain composition. These components effectively capture the key variability within the analyzed properties. Moreover, as the vector direction increases, the values of the corresponding traits rise, characterizing the samples accordingly, whereas movement in the opposite direction signifies a negative correlation among these traits.

The PCA score plot in Figure 8 illustrates the positioning of the red beetroot pestil samples along the PC1 and PC2 axes. The PCA loading plots (Figure 7) provide valuable insights into the principal components underlying the chemical variation observed among the red beetroot pestil samples. Variables contributing positively to the PC1 axis include phenolic compounds (chlorogenic acid, isoquercitrin, caffeic acid, p-coumaric acid, vanillin, etc.), antioxidant capacity indicators (CUPRAC, TPC, TFC, and DPPH), color parameters (L* and h*), and HMF. These variables were associated with the samples produced under the traditional control condition (Condition 1), as well as Conditions 2 and 7, indicating that these samples possess a richer content of biologically active compounds. In contrast, variables contributing negatively to the PC1 axis include color parameters (a, b, C*, and BI) and betalain pigments, such as betacyanins and betaxanthins, which were predominantly associated with samples processed under Conditions 9, 10, and 11. These conditions appear to enhance the color characteristics of the red beetroot pestil samples.

The PC2 axis is positively influenced by color-related parameters, such as BI, a, b, and C*, along with betalain pigment content. This indicates that samples processed under Conditions 9, 10, and 11 are positioned on the positive side of the PC2 axis and are characterized by more vivid colors and higher pigment concentrations. On the other hand, samples produced under Conditions 4, 5, and 8 are located on the negative side of the PC2 axis, suggesting that these samples have relatively lower color intensity. Similarly, samples produced under Condition 3, the traditional control condition (Condition 6), and Condition 12 are also positioned on the negative side of PC2 and are characterized not only by lower color parameter values but also by higher contents of o-salicylic acid, calcium (Ca), phosphorus (P), iron (Fe), magnesium (Mg), and zinc (Zn).

Overall, the PCA analysis reveals that different pretreatment conditions have significant effects on both the biologically active compound profiles and color attributes of the red beetroot pestil samples. In particular, phenolic compounds and betalain pigments demonstrate high sensitivity to the applied processing conditions.

#### 3.9.3. Hierarchical Cluster Analysis

**Figure 9:**
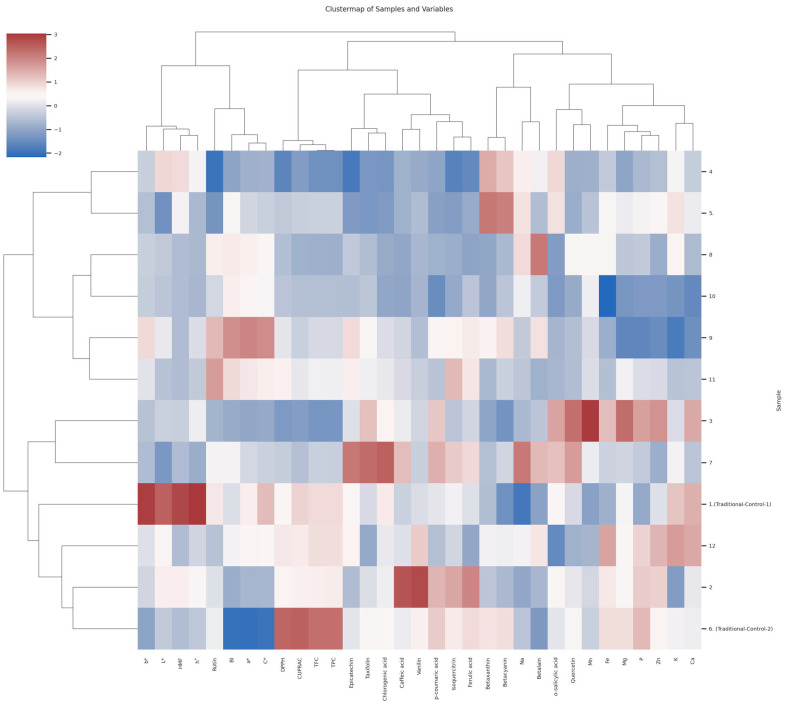
Cluster map of red beetroot pestils.

Hierarchical cluster analysis (HCA) is a systematic classification method used to identify natural groupings among the examined samples based on measured characteristics. In this technique, the data are first standardized, and then a dendrogram is generated through hierarchical clustering to reflect the similarities and differences among the samples [110]. Figure 9 presents hierarchical cluster analysis (HCA) conducted on the variables in the dataset, with the results visualized using a cluster map. This visualization clusters both the samples (rows) and variables (columns) simultaneously, enabling a more comprehensive evaluation of the data’s multivariate structure. Beyond a simple correlation matrix, it assesses both sample-level similarities and variable-based groupings, visually and statistically. Red beetroot pestils were grouped into five major clusters. The first cluster comprised Conditions 1 (Traditional Control 1), 2, 6 (Traditional Control 2), and 12; the second cluster included Conditions 3 and 7; the third cluster comprised Conditions 9 and 11; the fourth cluster included Conditions 8 and 10; and the fifth cluster comprised Conditions 4 and 5. These results clearly demonstrate the chemical differences among the pestils depending on the applied processing techniques. The close clustering of the traditionally processed samples (Conditions 1 and 6) indicates high similarity in their compositional profiles. In contrast, the separation of the pretreated samples (Conditions 4, 5, 8, 9, 10, and 11) into different clusters suggests that these processing conditions had a substantial impact, particularly on color parameters and betalain pigments. This distinction is also consistent with the results of the PCA.

Three main groups emerged when analyzing the clustering of all variables. The first major group included HMF, rutin, and color parameters (L*, a*, b*, C*, h°, and BI). The second group consisted of antioxidant parameters (TPC, TFC, CUPRAC, and DPPH) as well as individual phenolic compounds, such as epicatechin, taxifolin, chlorogenic acid, caffeic acid, vanillin, p-coumaric acid, isoquercitrin, and ferulic acid. The third group comprised mineral components (Mg, Fe, Na, Zn, Ca, P, Mn, and K), betalain pigments (betalain, betaxanthin, and betacyanin), o-salicylic acid, and quercetin. Upon more detailed examination of the graph, it is observed that HMF, rutin, and the color parameters in the first major group clustered closely based on Euclidean distances using the average linkage method. This finding supports the notion that thermal processing-induced color changes and Maillard reaction products emerge concurrently.

In conclusion, these findings highlight that multivariate statistical techniques, such as HCA and PCA, are effective tools for the evaluation of bioactive compounds, pigment profiles, mineral content, and thermal processing indicators, like HMF. Moreover, they are valuable for classifying the effects of different processing conditions on sample profiles.

## 4. Conclusions

This study evaluated the quality parameters of red beet (*Beta vulgaris* L.) pestil products produced using both conventional and innovative pretreatment methods. The results focused on the beneficial effects of innovative techniques, such as thermosonication and microwave treatments, in preserving the bioactive compounds of red beet. Both methods resulted in increased antioxidant capacity, phenolic compounds, and betalain content compared to conventional processing, while also improving physicochemical properties, including color and brix. Microwave treatment reduced nutrient losses through rapid energy transfer, whereas thermosonication enhanced the stability of food components and shortened the processing time. Moreover, their hybrid application with a decreased concentration period reduced HMF to the LOQ level.

Thermosonication and microwave pretreatments have shown the potential to enhance food quality, safety, and shelf-life; therefore, they have a wide applicability in food processing. However, the pathways for scaling-up these methods should focus on challenges related to heating uniformity, energy input and efficiency, processing time, and the complexity of the system. Solutions, such as integrating ultrasonic reactors and heated ultrasound modules, could address these challenges.

Novel pretreatment technologies, such as pulsed electric field (PEF), high hydrostatic pressure (HHP), microwave, ohmic heating, and radiation, have been proposed to enhance production performance. Despite their potential, these technologies are often more costly in terms of capital investment, energy, unit operations, and labor. Additionally, they tend to have a higher environmental impact, contributing to greenhouse gas emissions and global warming, compared to traditional methods. Nevertheless, they may be preferred in industrial applications due to their higher efficiency and shorter pretreatment durations.

Chemometric analyses, including PCA and Pearson correlation, were applied to differentiate sample groups and identify key contributors to antioxidant capacity. The analysis revealed strong associations between phenolic content, antioxidant activity, mineral composition, and color parameters, highlighting the sensitivity of bioactive components to specific pretreatment conditions.

Additionally, the bioactive compounds in red beet, including antioxidants, betanin, and flavonoids, along with its health-promoting properties, underscore its potential not only for fresh consumption but also as a valuable ingredient in processed food products. The functional properties of red beet support its application as both a healthy snack and a functional food ingredient. These findings suggest that the adoption of innovative pretreatment methods in pestil production can significantly enhance nutritional quality and preserve bioactive compounds.

Furthermore, the red beetroot pestils developed in this study offer promising potential as health-oriented snack products. Future studies should explore consumer acceptability, the in vivo bioavailability of minerals and phenolic compounds, and the product’s shelf-life under various storage conditions.

In conclusion, red beet-based products can be optimized for higher nutritional value and improved functional properties through the implementation of innovative processing techniques. This study provides a foundational framework for future research aimed at expanding the functional food potential of red beet and its application within the food industry. Moreover, the findings emphasize the critical role of such processing methods in improving product quality and maintaining nutritional integrity in food production.

## Figures and Tables

**Table 1:** Pretreatment conditions.

Trial/Conditions Number	1st Step of Pretreatment (Boiling) (Duration of the Process)	3rd Step of Pretreatment (Concentration) ^6^
		Final Degrees of Brix (°Bx) (Duration of the Process)
1. (Traditional Control 1)	Open vessel (15 min) ^1,4^	40 °Bx (120 min)
2.	[Thermosonication (30 min)] ^2,4^	40 °Bx (78 min)
3.	[Thermosonication (45 min)] ^2,4^	40 °Bx (58 min)
4.	[Thermosonication ^2^ (30 min) + microwave ^3^ (10 min)] ^4^	40 °Bx (60 min)
5.	[Thermosonication ^2^ (45 min) + microwave ^3^ (10 min)] ^4^	40 °Bx (30 min)
6. (Traditional Control 2)	Open vessel (15 min) ^1,4^	20 °Bx (78 min)
7	[Thermosonication (30 min)] ^2,4^	20 °Bx (27 min)
8	[Thermosonication (45 min)] ^2,4^	20 °Bx (6 min)
9	[Thermosonication ^2^ (30 min) + microwave ^3^ (10 min)] ^4^	20 °Bx (6 min)
10	[Thermosonication ^2^ (45 min) + microwave ^3^ (10 min)] ^4^	20 °Bx (4 min)
11	[Thermosonication^,^(45 min)] ^2,5^	not applicable
12	[Thermosonication ^2^ (45 min) + microwave ^3^ (10 min)] ^5^	not applicable

^1^ Heat treatment at 95 °C in an open vessel at atmospheric pressure. ^2^ Thermosonication at a frequency of 35 kHz and a temperature of 80 °C. ^3^ Microwave power: 360 W. ^4^ Blending together with the pretreatment water. ^5^ After draining the pretreatment water, the remaining fruits and vegetables were blended into a purée with °Bx. ^6^ Heat treatment at 100–103 °C in an open vessel at atmospheric pressure.

**Table 2:** Sensorial rating test and quality parameters.

Quality Parameter	Score
***Color***	
Characteristic color of red beetroot pestil, no caramelization	4
Slight browning in color, no caramelization	3
Dark brown color, caramelization present	2
Excessive caramelization due to burning	1
***Appearance***	
Transparent, uniform thickness	4
Matte, uniform thickness	3
Thickness is not uniform	2
Thickness is not uniform, matte, presence of clumping and irregular particles	1
***Taste and Aroma***	
Characteristic aromatic taste and aroma of red beetroot pestil.	4
Characteristic red beetroot aroma and taste, no foreign taste or odor	3
Characteristic red beetroot aroma and taste, with a very slight foreign fruit taste	2
Presence of foreign taste and odor	1

**Table 3:** Hedonic sensory test results.

Conditions	Color	Appearance	Taste	Odor	Beetroot-Specific Aroma	Beetroot-Specific Odor	Chewability	Texture	Overall Acceptability
1. (Traditional Control 1)	5.5 ± 0.9 ^b^	6.0 ± 1.6 ^c^	6.1 ± 1.3 ^abc^	6.1 ± 1.3 ^abc^	6.4 ± 1.2 ^ab^	6.2 ± 1.2 ^a^	6.7 ± 1.1 ^ab^	7.4 ± 0.9 ^a^	6.2 ± 1.1 ^ab^
2.	7.1 ± 1.0 ^ab^	7.5 ± 0.7 ^abc^	5.4 ± 0.5 ^c^	5.5 ± 0.5 ^c^	6.2 ± 1.1 ^ab^	6.7 ± 0.7 ^a^	5.0 ± 0.8 ^c^	6.5 ± 0.7 ^a^	6.5 ± 0.7 ^ab^
3.	6.5 ± 0.7 ^ab^	6.2 ± 1.1 ^bc^	5.2 ± 0.7 ^c^	6.4 ± 1.2 ^abc^	6.7 ± 0.7 ^ab^	6.7 ± 0.7 ^a^	5.4 ± 0.7 ^bc^	7.2 ± 0.9 ^a^	6.4 ± 1.2 ^ab^
4.	7.4 ± 1.3 ^a^	6.8 ± 1.0 ^abc^	7.1 ± 0.6 ^ab^	7.2 ± 0.9 ^ab^	7.0 ± 1.0 ^a^	6.8 ± 1.0 ^a^	7.1 ± 0.6 ^a^	7.4 ± 1.3 ^a^	7.4 ± 1.3 ^a^
5.	7.4 ± 1.3 ^a^	7.7 ± 0.9 ^abc^	7.2 ± 1.2 ^a^	6.4 ± 1.2 ^abc^	6.5 ± 0.9 ^ab^	6.4 ± 1.2 ^a^	7.4 ± 1.3 ^a^	7.4 ± 1.3 ^a^	7.1 ± 1.0 ^ab^
6. (Traditional Control 2)	7.2 ± 1.1 ^ab^	7.4 ± 0.9 ^abc^	5.5 ± 0.5 ^bc^	5.7 ± 0.4 ^bc^	5.8 ± 0.6 ^ab^	6.1 ± 0.9 ^a^	6.0 ± 0.8 ^abc^	6.8 ± 1.0 ^a^	6.2 ± 1.1 ^ab^
7	7.7 ± 0.9 ^a^	7.2 ± 0.7 ^abc^	7.4 ± 0.9 ^a^	7.4 ± 0.9 ^a^	7.4 ± 0.9 ^a^	7.0 ± 0.5 ^a^	7.5 ± 0.9 ^a^	7.1 ± 0.6 ^a^	7.5 ± 0.4 ^a^
8	7.5 ± 0.9 ^a^	7.2 ± 0.7 ^abc^	6.2 ± 1.1 ^abc^	6.4 ± 0.5 ^abc^	7.1 ± 0.6 ^a^	7.0 ± 0.5 ^a^	7.1 ± 0.6 ^a^	7.2 ± 0.7 ^a^	7.0 ± 0.5 ^ab^
9	8.1 ± 0.6 ^a^	8.1 ± 0.6 ^a^	6.7 ± 0.7 ^abc^	7.1 ± 0.6 ^abc^	7.0 ± 0.5 ^a^	6.8 ± 1.0 ^a^	6.1 ± 0.9 ^abc^	7.1 ± 0.6 ^a^	6.7 ± 0.7 ^ab^
10	8.1 ± 0.6 ^a^	8.0 ± 0.5 ^ab^	6.5 ± 0.5 ^abc^	6.5 ± 0.5 ^abc^	6.8 ± 1.0 ^ab^	6.7 ± 0.4 ^a^	7.0 ± 0.5 ^ab^	7.0 ± 0.5 ^a^	7.0 ± 0.5 ^ab^
11	8.1 ± 1.1 ^a^	7.7 ± 0.9 ^abc^	7.4 ± 0.7 ^a^	6.5 ± 0.6 ^abc^	7.4 ± 0.7 ^a^	7.4 ± 0.7 ^a^	7.5 ± 0.9 ^a^	7.5 ± 0.9 ^a^	7.5 ± 0.9 ^a^
12	7.0 ± 1.3 ^ab^	6.2 ± 1.3 ^bc^	5.2 ± 0.9 ^c^	5.5 ± 0.7 ^c^	5.2 ± 0.9 ^b^	5.8 ± 0.6 ^a^	5.0 ± 0.8 ^c^	6.2 ± 1.1 ^a^	5.9 ± 0.6 ^b^

Different letters in the columns represent statistically significant differences (*p* < 0.05).

**Table 4:** Color parameters and HMF contents of red beetroot.

Conditions	L*	a*	b*	C*	h°	BI	HMF (mg/kg)
1. (Traditional Control 1)	30.80 ± 0.02 ^a^	10.83 ± 0.11 ^b^	7.83 ± 0.06 ^a^	13.35 ± 0.11 ^b^	35.84 ± 0.23 ^a^	26.47 ^e^	282.01 ± 5.85 ^a^
2.	26.06 ± 0.13 ^bc^	7.42 ± 0.08 ^d^	2.66 ± 0.05 ^d^	7.87 ± 0.10 ^e^	19.64 ± 0.07 ^b^	20.54 ^f^	100.07 ± 4.13 ^c^
3.	23.85 ± 0.24 ^ef^	6.80 ± 0.09 ^d^	2.14 ± 0.10 ^e^	7.19 ± 0.32 ^e^	18.37 ± 0.17 ^c^	20.44 ^f^	25.73 ± 0.92 ^e^
4.	26.83 ± 0.15 ^b^	7.17 ± 0.07 ^d^	2.47 ± 0.05 ^d^	7.67 ± 0.11 ^e^	18.98 ± 0.51 ^bc^	19.30 ^g^	120.05 ± 0.2 ^b^
5.	20.98 ± 0.49 ^g^	8.77 ± 0.24 ^c^	2.05 ± 0.07 ^e^	9.00 ± 0.24 ^d^	13.15 ± 0.12 ^g^	29.01 ^c^	70.71 ± 0.50 ^d^
6. (Traditional Control 2)	23.60 ± 0.33 ^f^	3.99 ± 0.14 ^e^	1.22 ± 0.16 ^f^	4.29 ± 0.38 ^f^	14.75 ± 0.24 ^f^	12.39	2.23 ± 0.40 ^f^
7	21.29 ± 0.56 ^g^	8.82 ± 0.64 ^c^	1.98 ± 0.12 ^e^	9.09 ± 0.64 ^d^	11.90 ± 0.52 ^h^	28.73 ^d^	<LOQ
8	23.56 ± 0.39 ^f^	10.77 ± 0.28 ^b^	2.48 ± 0.07 ^d^	10.96 ± 0.14 ^c^	13.06 ± 0.18 ^g^	31.52 ^b^	0.31 ± 0.06 ^f^
9	24.82 ± 0.09 ^de^	14.41 ± 0.11 ^a^	4.39 ± 0.04 ^b^	15.07 ± 0.10 ^a^	16.96 ± 0.22 ^d^	39.75 ^a^	<LOQ
10	23.39 ± 0.06 ^f^	10.50 ± 0.10 ^b^	2.42 ± 0.05 ^d^	10.77 ± 0.11 ^c^	12.96 ± 0.16 ^g^	30.99 ^c^	<LOQ
11	23.22 ± 0.18 ^f^	11.06 ± 0.03 ^b^	3.03 ± 0.12 ^c^	11.48 ± 0.08 ^c^	14.96 ± 0.15 ^f^	32.96 ^b^	1.09 ± 0.10 ^f^
12	25.74 ± 0.71 ^cd^	10.53 ± 0.11 ^b^	2.95 ± 0.07 ^c^	10.96 ± 0.10 ^c^	16.01 ± 0.14 ^d^	28.62 ^d^	<LOQ

Different letters in the columns represent statistically significant differences (*p* < 0.05).

**Table 5:** Mineral compositions (mg/kg).

Conditions	Na (mg/kg)	Mg (mg/kg)	P (mg/kg)	K (mg/kg)	Ca (mg/kg)	Mn (mg/kg)	Fe (mg/kg)	Zn (mg/kg)
1. (Traditional Control 1)	850.01 ± 0.01 ^g^	397.95 ± 8.74 ^c^	592.80 ± 26.70 ^def^	7280.10 ± 69.70 ^ab^	113.33 ± 1.44 ^a^	1.24 ± 0.05 ^h^	5.39 ± 0.05 ^cd^	2.95 ± 0.04 ^de^
2.	1124.10 ± 34.70 ^f^	402.99 ± 3.62 ^c^	913.42 ± 13.76 ^a^	4308.00 ± 19.61 ^cde^	91.75 ± 1.34 ^b^	1.58 ± 0.01 ^fg^	7.69 ± 0.54 ^ab^	4.07 ± 0.01 ^b^
3.	1213.50 ± 20.40 ^ef^	545.33 ± 3.60 ^a^	1006.30 ± 18.10 ^a^	5725.98 ± 10.87 ^cde^	114.52 ± 1.81 ^a^	3.15 ± 0.06 ^a^	8.50 ± 0.53 ^a^	5.01 ± 0.05 ^a^
4.	1589.30 ± 28.20 ^bc^	302.08 ± 0.25 ^e^	638.74 ± 3.31 ^cde^	6208.70 ± 45.20 ^abcd^	84.85 ± 1.30 ^c^	1.46 ± 0.02 ^g^	6.09 ± 0.06 ^bc^	2.31 ± 0.24 ^c^
5.	1636.50 ± 59.10 ^b^	387.9 ± 14.9 ^c^	790.10 ± 72.70 ^b^	6792.00 ± 142.00 ^ab^	92.95 ± 2.66 ^b^	2.10 ± 0.09 ^c^	7.13 ± 0.04 ^bc^	3.41 ± 0.12 ^cd^
6. (Traditional Control 2)	1271.46 ± 2.18 ^e^	437.04 ± 2.14 ^b^	953.15 ± 12.72 ^a^	6126.06 ± 10.3^2 abcd^	93.24 ± 1.45 ^b^	1.76 ± 0.04 ^ef^	7.91 ± 0.37 ^ab^	3.55 ± 0.25 ^c^
7	2047.66 ± 12.36 ^a^	360.42 ± 0.75 ^d^	687.70 ± 42.40 ^bcd^	6143.80 ± 33.90 ^abcd^	82.84 ± 0.57 ^c^	2.42 ± 0.01 ^b^	6.28 ± 0.17 ^bc^	1.96 ± 0.08 ^fg^
8	1662.89 ± 4.10 ^b^	344.44 ± 3.79 ^d^	684.62 ± 7.43 ^bcd^	6439.90 ± 34.80 ^abc^	79.82 ± 0.50 ^c^	1.86 ± 0.01 ^de^	7.14 ± 0.02 ^abc^	1.94 ± 0.03 ^fg^
9	1305.09 ± 8.08 ^e^	259.34 ± 1.37 ^f^	487.30 ± 16.20 ^f^	3485.38 ± 6.72 ^e^	67.10 ± 0.68 ^d^	2.04 ± 0.03 ^cd^	5.35 ± 1.43 ^cd^	1.27 ± 0.08 ^h^
10	1469.55 ± 3.57 ^d^	280.21 ± 0.794 ^ef^	547.72 ± 1.50 ^ef^	4107.48 ± 13.84 ^de^	64.96 ± 2.35 ^d^	2.06 ± 0.02 ^c^	3.35 ± 0.06 ^d^	1.56 ± 0.13 ^gh^
11	1283.20 ± 15.30 ^e^	393.38 ± 5.41 ^c^	730.16 ± 8.26 ^bc^	5182.80 ± 29.80 b^cde^	82.90 ± 1.64 ^c^	2.12 ± 0.05 ^c^	5.82 ± 0.10 ^bc^	2.81 ± 0.01 ^e^
12	1488.70 ± 49.30 ^cd^	403.42 ± 13.03 ^c^	898.64 ± 6.55 ^a^	7955.00 ± 305.00 ^a^	114.48 ± 0.98 ^a^	1.48 ± 0.04 ^g^	9.03 ± 0.71 ^a^	4.47 ± 0.01 ^b^

Different letters in the columns represent statistically significant differences (*p* < 0.05).

**Table 6:** Total betalain content (mg/kg).

Conditions	Betaxanthin (mg/kg)	Betacyanin (mg/kg)	Total Betalain (mg/kg)
1. (Traditional Control 1)	145.86 ± 7.71 ^d^	168.53 ± 9.20 ^f^	314.40 ± 16.91 ^ef^
2	151.76 ± 16.13 ^cd^	208.27 ± 23.59 ^ef^	360.03 ± 39.72 ^ef^
3	128.68 ± 13.02 ^d^	171.64 ± 15.69 ^f^	300.32 ± 28.71 ^f^
4	229.00 ± 23.36 ^a^	306.01 ± 33.60 ^b^	535.01 ± 56.97 ^b^
5.	257.10 ± 18.52 ^a^	355.90 ± 13.22 ^a^	613.00 ± 31.70 ^a^
6. (Traditional Control 2)	200.07 ± 5.25 ^b^	288.59 ± 8.45 ^bc^	488.66 ± 13.69 ^bc^
7	147.98 ± 5.34 ^d^	234.70 ± 6.55 ^de^	382.68 ± 11.89 ^de^
8	131.64 ± 3.49 ^d^	209.80 ± 2.06 ^def^	341.44 ± 1.44 ^ef^
9	192.20 ± 29.60 ^b^	289.10 ± 61.60 ^bc^	481.30 ± 91.21 ^bc^
10	130.76 ± 3.89 ^d^	220.17 ± 12.02 ^de^	350.93 ± 15.91 ^de^
11	143.76 ± 11.14 ^d^	229.04 ± 20.80 ^de^	372.80 ± 31.94 ^ef^
12	179.41 ± 7.22 ^bc^	255.37 ± 10.08 ^cd^	434.79 ± 17.30 ^bc^

Different letters in the columns represent statistically significant differences (*p* < 0.05).

**Table 7:** Changes in TAC, TPC, and TFC for different pretreatment conditions (mg/kg).

Conditions	TAC		TPC (mg GAE/100 g DM)	TFC (mg RE/100 g DM)
	CUPRAC (mg TE/100 g DM)	DPPH (mg TE/100 g DM)		
1. (Traditional Control 1)	587.48 ± 42.90 ^b^	30.85 ± 2.84 ^bc^	220.60 ± 9.63 ^b^	365.08 ± 15.71 ^b^
2	515.38 ± 11.96 ^c^	31.98 ± 1.06 ^b^	205.46 ± 3.80 ^c^	340.48 ± 6.20 ^c^
3	183.90 ± 4.01 ^h^	17.28 ± 0.88 ^e^	82.01 ± 2.03 ^i^	138.62 ± 3.31 ^i^
4	182.07 ± 26.48 ^h^	14.05 ± 0.46 ^f^	76.26 ± 2.37 ^i^	128.19 ± 3.86 ^i^
5.	347.30 ± 10.21 ^e^	24.65 ± 0.50 ^d^	149.13 ± 1.55 ^ef^	248.13 ± 2.53 ^ef^
6. (Traditional Control 2)	870.78 ± 25.05 ^a^	48.18 ± 0.76 ^a^	313.17 ± 10.63 ^a^	517.46 ± 17.35 ^a^
7	304.84 ± 40.50 ^ef^	24.93 ± 2.57 ^d^	146.43 ± 5.82 ^f^	245.47 ± 9.49 ^f^
8	255.29 ± 17.34 ^g^	22.94 ± 0.72 ^d^	113.11 ± 2.15 ^h^	190.96 ± 3.51 ^h^
9	348.05 ± 7.98 ^e^	28.20 ± 0.63 ^c^	157.54 ± 2.30 ^e^	263.66 ± 3.76 ^e^
10	301.72 ± 10.37 ^f^	23.69 ± 0.95 ^d^	130.75 ± 4.83 ^g^	218.78 ± 7.88 ^g^
11	420.81 ± 19.47 ^d^	32.46 ± 0.88 ^b^	178.90 ± 3.05 ^d^	299.12 ± 4.97 ^d^
12	530.50 ± 19.16 ^c^	33.68 ± 2.26 ^b^	218.34 ± 5.76 ^b^	362.96 ± 9.40 ^b^

Different letters in the columns represent statistically significant differences (*p* < 0.05).

**Table 8:** Method parameters used for the determination of polyphenols.

Compound	Retention Time (RT)	Ionization Mode	Mass (*m*/*z*)	Main Fragment (*m*/*z*)	Other Fragmental Ions (*m*/*z*)
***Phenolic acids and other polyphenols***					
Chlorogenic acid	2.01	ESI-	353.1	191.1	110.9
Ferulic acid	3.79	ESI-	192.9	134.1	178.1
Caffeic acid	2.67	ESI-	178.8	135.1	89.2
o-salicylic acid	5.06	ESI-	137.1	93.0	75.0; 65.0
p-coumaric acid	3.54	ESI-	163.1	119.1	146.0; 117.1; 93.0; 65.0; 41.0
Vanillin	3.55	ESI-	151.0	136.1	92.1
***Flavonoid***					
Isoquercitrin	3.45	ESI-	463.1	300.0	271.0
Epicatechin	2.44	ESI-	289.1	245.1	205.1
Quercetin	5.95	ESI-	301.0	151.2	179.0
Rutin	3.10	ESI-	609.1	300.0	271.1
Taxifolin	3.87	ESI-	303.1	285.1	125.0

**Table 9:** Changes in the flavonoid content of red beetroot pestils (µg/kg).

Conditions	Epicatechin	Rutin	Isoquercitrin	Taxifolin	Quercetin
1. (Traditional Control 1)	2693.17 ± 109.03 ^c^	14.49 ± 0.36 ^bc^	119.10 ± 1.31 ^cd^	2.95 ± 0.02 ^cd^	13.59 ± 0.35 ^d^
2	1771.29 ± 165.76 ^e^	12.74 ± 0.62 ^def^	161.55 ± 8.22 ^a^	3.00 ± 0.36 ^cd^	15.59 ± 0.60 ^d^
3	2307.19 ± 109.48 ^d^	10.84 ± 0.25 ^gh^	96.43 ± 2.58 ^ef^	4.02 ± 0.24 ^b^	38.59 ± 1.93 ^a^
4	651.88 ± 0.65 ^g^	7.78 ± 0.36 ^i^	56.29 ± 2.27 ^h^	2.01 ± 0.06 ^f^	5.87 ± 0.86 ^g^
5.	1240.70 ± 98.96 ^f^	9.33 ± 0.07 ^h^	73.67 ± 1.25 ^g^	2.01 ± 0.02 ^f^	5.43 ± 0.64 ^g^
6. (Traditional Control 2)	2371.00 ± 182.25 ^d^	13.16 ± 1.42 ^cde^	132.47 ± 10.24 ^bc^	3.39 ± 0.12 ^c^	18.21 ± 2.06 ^c^
7	4306.23 ± 103.54 ^a^	13.43 ± 0.24 ^cde^	146.35 ± 0.41 ^ab^	4.94 ± 0.20 ^a^	32.21 ± 0.85 ^b^
8	1978.33 ± 168.21 ^e^	14.26 ± 0.39 ^cd^	83.87 ± 1.35 ^fg^	2.56 ± 0.24 ^de^	18.11 ± 0.84 ^c^
9	3097.76 ± 16.57 ^b^	15.96 ± 1.67 ^ab^	128.42 ± 12.08 ^c^	3.38 ± 0.22 ^c^	8.68 ± 1.00 ^e^
10	1807.34 ± 138.79 ^e^	12.33 ± 0.85 ^efg^	80.69 ± 0.23 ^fg^	2.69 ± 0.17 ^de^	5.00 ± 0.18 ^g^
11	2882.42 ± 141.92 ^bc^	16.89 ± 0.05 ^a^	153.74 ± 21.61 ^a^	3.20 ± 0.42 ^c^	8.52 ± 0.80 ^ef^
12	2812.91 ± 5.97 ^c^	11.46 ± 0.01 ^fg^	105.47 ± 0.29 ^de^	2.28 ± 0.18 ^ef^	6.40 ± 0.21 ^fg^

Different letters in the columns represent statistically significant differences (*p* < 0.05).

**Table 10:** Changes in the phenolic acids and other polyphenols content of red beetroot pestils (µg/kg).

Conditions	Chlorogenic Acid	Ferulic Acid	Caffeic Acid	o-Salicylic Acid	p-Coumaric Acid	Vanillin
1. (Traditional Control 1)	56,274.93 ± 719.86 ^b^	259.72 ± 4.68 ^c^	19.93 ± 0.66 ^e^	42.51 ± 0.59 ^cd^	34.14 ± 1.95 ^b^	234.86 ± 6.03 ^d^
2	46,783.23 ± 414.76 ^d^	432.22 ± 13.82 ^a^	54.93 ± 2.05 ^a^	42.86 ± 2.48 ^cd^	48.93 ± 3.23 ^a^	554.20 ± 36.88 ^a^
3	53,430.91 ± 477.30 ^bc^	245.37 ± 11.01 ^cd^	24.89 ± 0.62 ^c^	55.70 ± 0.08 ^a^	46.43 ± 0.35 ^a^	22.83 ± 1.53 ^d^
4	16,351.71 ± 329.13 ^g^	128.70 ± 0.76 ^f^	13.47 ± 0.99 ^fg^	47.83 ± 0.37 ^b^	21.86 ± 0.27 ^d^	143.64 ± 1.79 ^g^
5.	19,001.83 ± 192.20 ^g^	184.90 ± 7.45 ^e^	13.63 ± 0.61 ^f^	46.74 ± 0.28 ^bc^	20.56 ± 2.96 ^de^	177.14 ± 0.29 ^f^
6. (Traditional Control 2)	51,236.94 ± 383.32 ^c^	335.03 ± 5.99 ^b^	25.01 ± 0.29 ^c^	38.41 ± 0.83 ^d^	45.74 ± 3.12 ^a^	310.37 ± 19.14 ^c^
7	92,638.67 ± 1077.40 ^a^	337.86 ± 14.74 ^b^	37.88 ± 0.20 ^b^	51.44 ± 4.76 ^ab^	49.84 ± 5.48 ^a^	209.86 ± 6.00 ^de^
8	25,139.23 ± 1928.66 ^f^	172.86 ± 6.31 ^e^	11.33 ± 0.21 ^gh^	24.43 ± 0.39 ^f^	24.44 ± 0.28 ^cd^	167.57 ± 5.77 ^fg^
9	41,683.02 ± 2151.84 ^e^	317.55 ± 12.90 ^b^	20.83 ± 0.48 ^e^	29.55 ± 3.55 ^e^	38.66 ± 0.10 ^b^	181.45 ± 2.93 ^ef^
10	23,426.25 ± 490.18 ^f^	222.84 ± 1.44 ^d^	10.83 ± 0.01 ^h^	23.97 ± 2.54 ^f^	16.59 ± 2.44 ^e^	164.54 ± 5.80 ^fg^
11	45,136.42 ± 3556.85 ^d^	324.02 ± 35.34 ^b^	21.82 ± 2.51 ^de^	29.95 ± 1.64 ^e^	27.98 ± 2.27 ^c^	211.68 ± 25.36 ^de^
12	45,449.35 ± 1449.61 ^d^	179.25 ± 1.79 ^e^	23.08 ± 0.04 ^cd^	20.62 ± 3.17 ^f^	27.1 ± 0.08 ^c^	362.36 ± 12.93 ^b^

Different letters in the columns represent statistically significant differences (*p* < 0.05).

**Table 11:** Variance ratios explained by the first seven principal components.

Variables	PC1	PC2	PC3	PC4	PC5	PC6	PC7
Eigenvalue	9.326	6.372	5.684	4.217	2.272	1.905	1.342
Percentage %	28.30	19.30	17.20	12.80	6.90	5.80	4.10
Cumulative	28.30	47.60	64.80	77.60	84.50	90.20	94.30
DPPH	0.597	0.368	−0.290	−0.596	0.147	−0.038	−0.201
CUPRAC	0.642	0.275	−0.480	−0.462	0.077	0.049	−0.208
TFC	0.628	0.349	−0.417	−0.508	0.125	0.065	−0.153
TPC	0.629	0.345	−0.422	−0.506	0.120	0.066	−0.153
Epicatechin	0.328	0.616	0.488	−0.001	0.359	0.323	−0.084
Rutin	0.135	0.875	0.199	−0.088	0.147	−0.175	−0.170
Isoquercitrin	0.658	0.624	0.229	−0.189	−0.093	0.007	0.154
Quercetin	0.538	−0.156	0.650	0.422	0.047	0.132	−0.155
Taxifolin	0.476	0.304	0.727	0.165	−0.090	0.255	−0.201
Caffeic acid	0.729	0.180	0.249	−0.040	−0.271	0.043	0.527
o-salicylic acid	0.368	−0.430	0.176	0.413	−0.451	0.464	−0.018
Vanillin	0.729	0.079	−0.172	−0.178	−0.064	−0.257	0.541
p-coumaric acid	0.802	0.172	0.396	0.091	−0.187	0.193	0.118
Ferulic acid	0.650	0.521	0.269	−0.200	−0.394	−0.064	0.130
Chlorogenic acid	0.635	0.372	0.444	0.147	0.125	0.436	−0.034
Na	−0.406	−0.317	0.569	−0.329	0.190	0.428	0.118
Mg	0.764	−0.396	0.050	0.292	0.237	−0.162	−0.171
P	0.752	−0.558	0.053	−0.102	0.199	−0.237	0.075
K	0.159	−0.378	−0.379	0.148	0.656	0.419	−0.071
Ca	0.606	−0.312	−0.362	0.434	0.427	0.080	0.044
Fe	0.597	−0.512	0.014	−0.070	0.454	−0.036	0.308
Zn	0.704	−0.483	−0.193	0.160	0.244	−0.245	0.147
Mn	0.186	−0.300	0.669	0.394	0.020	−0.410	−0.204
Betalain	−0.463	0.047	0.497	0.003	0.442	0.184	0.349
Betaxanthin	−0.206	−0.436	−0.413	−0.444	−0.149	0.431	0.138
Betacyanin	−0.339	−0.353	−0.184	−0.664	−0.115	0.358	0.091
HMF	0.116	0.104	−0.677	0.568	−0.256	0.287	0.061
L*	0.134	0.295	−0.685	0.512	−0.061	0.002	0.149
a*	−0.577	0.674	0.027	0.130	0.285	−0.081	0.206
b*	−0.050	0.623	−0.516	0.547	0.064	0.152	0.007
C*	−0.502	0.725	−0.103	0.245	0.261	−0.020	0.162
h°	0.246	0.311	−0.626	0.644	−0.104	0.137	−0.014
BI	−0.619	0.577	0.225	0.005	0.288	−0.056	0.162

## Data Availability

The original contributions presented in this study are included in the article. Further inquiries can be directed to the corresponding author.
